# Exploratory Multi-Platform Bioinformatic Analysis of MEAK7 and Its Molecular, Survival, and Immune Associations in Pancreatic Ductal Adenocarcinoma

**DOI:** 10.3390/ijms27177610

**Published:** 2026-08-25

**Authors:** Meltem Uyaner Kan, Durmus Ayan

**Affiliations:** 1 Medical Biochemistry, Nigde Omer Halisdemir University Research and Training Hospital, Nigde 51000, Türkiye; muyaner@gmail.com; 2 Medical Biochemistry, Faculty of Medicine, Nigde Omer Halisdemir University, Nigde 51240, Türkiye; 3 Medical Biochemistry, Dr. Abdurrahman Yurtaslan Ankara Oncology Training and Research Hospital, Ankara 06200, Türkiye

**Keywords:** pancreatic ductal adenocarcinoma, *MEAK7*, *KIAA1609*, prognostic biomarker, survival, bioinformatic analysis

## Abstract

Pancreatic ductal adenocarcinoma (PDAC) is associated with high mortality owing to late diagnosis, aggressive tumor biology, and limited clinically useful early diagnostic and prognostic biomarkers. This study provides an exploratory evaluation of the expression profile, survival associations, co-expressed genes, functional association networks, and regulatory relationships of *MEAK7* (*KIAA1609/TLDC1*) in pancreatic cancer using multi-platform bioinformatic approaches. We conducted a comprehensive multi-platform bioinformatic analysis using multiple publicly available datasets and platforms, including TCGA/GTEx, GEO, GEPIA3, HPA, CPTAC/UALCAN, TNMplot, Kaplan–Meier Plotter, DoSurvive, TIMER 3.0, STRING, TargetScan, miRDB, ENCORI and lncRNADisease. Gene expression patterns, survival associations, immune cell infiltration and regulatory non-coding RNA networks were systematically investigated. A STRING-derived protein functional association network was evaluated. *MEAK7* expression patterns were additionally assessed in external GEO datasets, including GSE62165, GSE71729, and GSE183795. In summary, *MEAK7* expression was elevated in pancreatic cancer compared with normal pancreatic tissues, was detectably expressed across multiple pancreatic cancer cell lines, and was increased in patient-derived PDAC samples. ROC analysis showed that *MEAK7* expression discriminated PDAC tumor tissues from adjacent non-tumor pancreatic tissues, with an AUC of 0.795 (95% CI: 0.719–0.871) indicating exploratory transcriptomic discrimination rather than clinical diagnostic performance. Exploratory survival analyses showed that elevated *MEAK7* expression was associated with unfavorable overall survival and disease-free outcomes, while multivariable Cox analyses showed that this association persisted after adjustment for the covariates available within the analyzed dataset. GEO-based analyses supported increased *MEAK7* expression in early-stage pancreatic cancer samples, although metastatic expression patterns varied across datasets. Correlation and interaction analyses revealed positive associations between *MEAK7* and PDAC-related genes, including *CTTN*, *CDCP1*, *SMARCA4*, *SFN*, and *PALB2*, as well as high-confidence STRING functional associations with V-ATPase components and RNASEK. Furthermore, *MEAK7* was positively correlated with lncRNA UCA1 and negatively correlated with miR-582-5p. Collectively, these findings identify reproducible expression and survival-associated patterns involving MEAK7 across the analyzed datasets and provide a hypothesis-generating framework for further investigation of its potential biological role in PDAC. Experimental studies and prospectively characterized patient cohorts are required before any clinical, prognostic, diagnostic, or therapeutic implications can be established.

## 1. Introduction

Pancreatic cancer is the third leading cause of cancer-related mortality, with a reported five-year survival rate of 13% [1]. Considering all stages of the disease, the median overall survival duration was around four months. This limited survival time is attributed to asymptomatic progression, delayed diagnosis, and aggressive tumor nature. Consequently, pancreatic cancer is anticipated to become the second leading cause of cancer-related mortality by 2030 [2].

Innovative and effective strategies are imperative for the diagnosis and treatment of pancreatic cancer, a malignancy characterized by resistance to conventional chemotherapy, early and extensive metastatic spread, and a paucity of effective immunotherapeutic options [3,4]. With advancements in our understanding of tumor biology and molecular pathways, the identification of novel biomarkers has gained significance. In pancreatic ductal adenocarcinoma (PDAC), particular attention has been directed towards cellular signaling pathways that are pivotal in tumor proliferation, metabolic reprogramming, and regulation of metastatic potential. The most prevalent somatic mutation in PDAC is *KRAS*, which is present in over 90% of patients [5]. The phosphatidylinositol-3 kinase/protein kinase B/mammalian target of rapamycin (PI3K/Akt/mTOR) signaling pathway is one of the major signaling mechanisms activated downstream of *KRAS* and plays a critical role in the regulation of cellular growth, survival, motility, and metabolism. Alterations in the PI3K signaling pathway have been documented in nearly half of all cancers and in 60% of pancreatic cancer cases [6,7]. Elevated *mTOR* expression correlates with increased tumor cell proliferation. Targeting the mTOR pathway is being explored as a potential therapeutic strategy for the treatment of pancreatic cancer [6,8].

MEAK7 (mammalian EAK-7 or MTOR-associated protein, eak-7 homolog) is a recently identified protein that may regulate cell proliferation and migration in human cells by activating an alternative mTOR signaling pathway via S6K2 and 4E-BP1 [9,10]. In a study utilizing a PDAC organoid model, alternative mTOR complexes, including MEAK7 and mTOR G protein-coupled receptor kinase-interacting ArfGAP 1 (GIT1), were shown to play a role in the development of the PDAC fibrotic barrier. Furthermore, concurrent inhibition of MEAK7 and GIT1 decreased collagen I expression in pancreatic stellate cells and enhanced macromolecular permeability [11]. These findings suggest that MEAK7 plays a role in regulating the extracellular matrix within the PDAC stroma and may be relevant to stromal remodeling and drug permeability. However, the expression characteristics, clinical associations, and prognostic relevance of *MEAK7* in pancreatic cancer remain insufficiently characterized.

In recent years, high-throughput transcriptomic datasets combined with bioinformatics analyses have become valuable tools for elucidating the functional relevance and clinical implications of understudied genes in cancer [12,13,14]. While numerous bioinformatics investigations have explored potential biomarkers in PDAC, most have concentrated on well-established oncogenic pathways or frequently examined gene families [15]. Conversely, there has been limited focus on less-studied regulatory molecules that may play significant roles in tumor biology. *MEAK7* (*KIAA1609/TLDC1*), a relatively underexplored protein linked to mTOR signaling and cellular stress responses, has not been extensively characterized in the context of PDAC using multi-platform bioinformatic methodologies.

The Cancer Genome Atlas (TCGA) designates pancreatic adenocarcinoma as PAAD. As most tumors within the TCGA-PAAD cohort are pancreatic ductal adenocarcinoma (PDAC), this manuscript uses the term PAAD when referring to the TCGA dataset. Conversely, PDAC is used to describe the biological and clinical characteristics specific to this histological subtype.

In this study, we hypothesized that MEAK7 is aberrantly expressed in PDAC and is associated with biologically and clinically annotated features of the disease. To investigate this hypothesis, we performed an exploratory multi-platform bioinformatic analysis using publicly available transcriptomic, proteomic, and clinical datasets to characterize MEAK7 expression across normal, tumor, and metastatic tissues, examine its associations with survival and immune-cell infiltration, and explore its molecular functional association networks and regulatory non-coding RNA relationships. The study was designed as a hypothesis-generating investigation aimed at identifying reproducible molecular and clinical associations that may guide subsequent experimental and prospectively designed validation studies, rather than establishing diagnostic, prognostic, or therapeutic utility.

## 2. Results

### 2.1. Differential Gene and Protein Expression of MEAK7 in Pancreatic Adenocarcinoma (PAAD)

**Figure 1:**
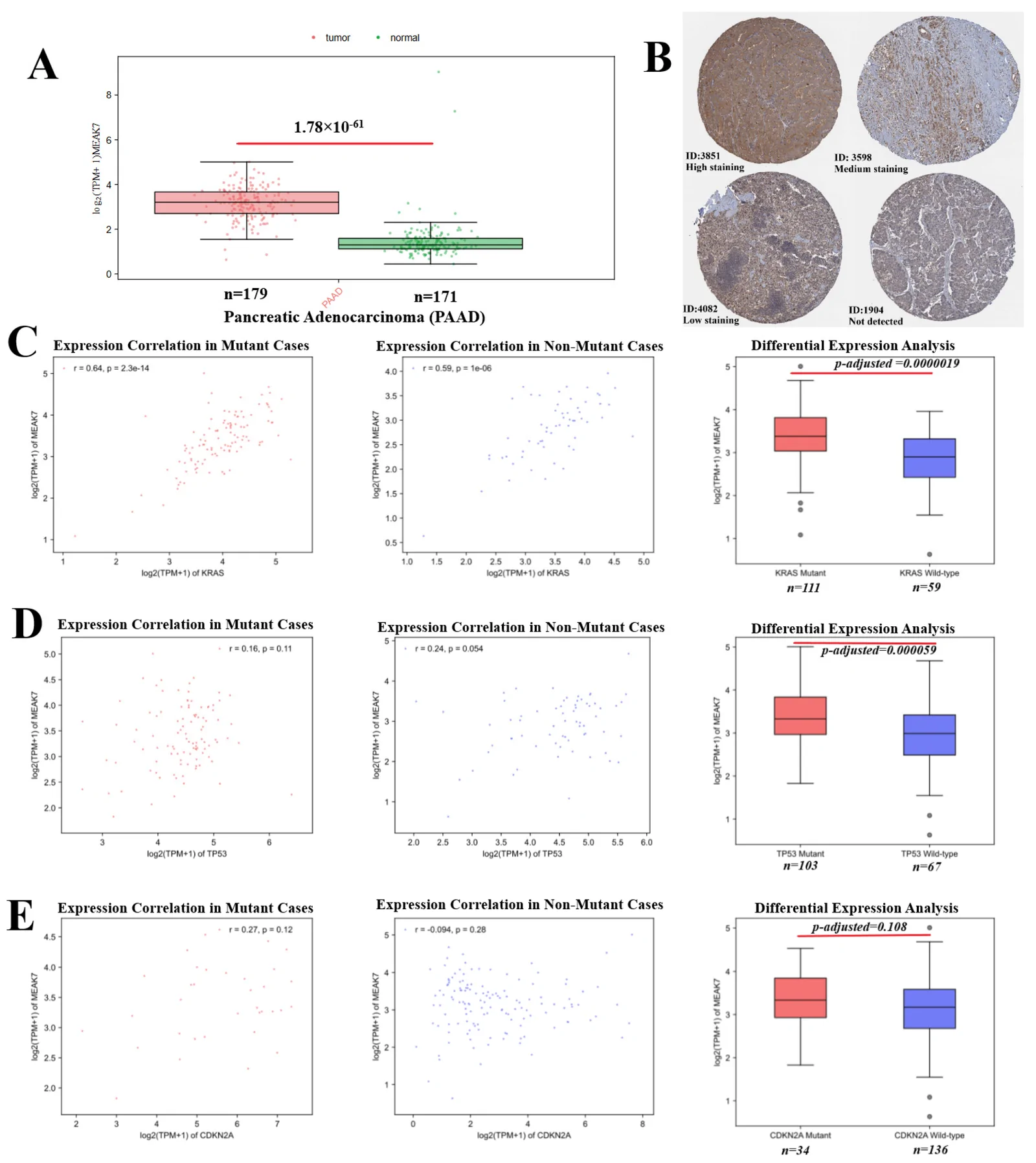
Expression profile of *MEAK7* in PDAC and its association with key oncogenic driver mutations. (**A**) *MEAK7* expression in TCGA-PAAD tumors and GTEx normal pancreas; (**B**) HPA immunohistochemical staining of MEAK7 protein; (**C**) *MEAK7–KRAS* associations by mutation status; (**D**) *MEAK7–TP53* associations by mutation status; (**E**) *MEAK7–CDKN2A* associations by mutation status.

The expression profile of *MEAK7* in PAAD was comprehensively evaluated at both the gene and protein levels. In transcriptomic analyses, *MEAK7* expression was comparatively examined between pancreatic tumor tissues (*n* = 179) and normal pancreatic tissues (*n* = 171). These analyses were performed using RNA-seq data from TCGA-PAAD tumor samples and GTEx normal pancreatic tissues using the GEPIA3 platform. The results showed that *MEAK7* expression levels were upregulated in tumor tissues compared to normal pancreatic tissues (adj*p* = 1.78 × 10^−61^) (Figure 1A). Immunohistochemical images obtained from the Human Protein Atlas (HPA) revealed the heterogeneous expression of MEAK7 in PAAD tissues. The samples exhibited a range of staining patterns, including high, medium, low, and undetectable staining levels. These findings were interpreted descriptively based on the HPA-provided annotations (Figure 1B). In analyses concerning *KRAS*, a positive and significant correlation was identified between *MEAK7* expression and *KRAS* expression (mutant cases: *r* = 0.64, adj*p* = 2.3 × 10^−14^; non-mutant cases: *r* = 0.59, adj*p* = 1 × 10^−6^). Additionally, *MEAK7* expression was significantly upregulated in *KRAS* mutant cases compared to that in the *KRAS* wild-type group (*p*-adjusted = 0.000019) (Figure 1C). In the correlation analyses with *TP53*, no significant correlation was observed between *MEAK7* expression and *TP53* expression in either the mutant or non-mutant cases (*r* = 0.16, adj*p* = 0.11 and *r* = 0.24, adj*p* = 0.054, respectively). However, *MEAK7* expression was significantly elevated in *TP53* mutant tumors compared to *TP53* wild-type tumors (*p*-adjusted = 0.000059) (Figure 1D). No significant correlation was detected between *MEAK7* and *CDKN2A* in either the mutant or non-mutant cases (*r* = 0.27, adj*p* = 0.12 and *r* = −0.094, adj*p* = 0.28, respectively). Furthermore, there was no statistically significant difference in *MEAK7* expression between the *CDKN2A* mutant and wild-type groups (*p*-adjusted = 0.108) (Figure 1E).

### 2.2. Exploratory ROC-Based Tissue Expression Discrimination Analysis

An exploratory receiver operating characteristic (ROC) analysis was performed on the GSE62452 cohort, which included 69 primary PDAC tumor samples and 61 adjacent non-tumor pancreatic tissue samples. This analysis aimed to assess the tissue expression discrimination of *MEAK7* in comparison with the principal PDAC driver genes *KRAS*, *TP53*, *CDKN2A*, and *SMAD4*. Gene-specific cut-offs were determined using the Youden index and evaluated within the same cohort, rendering the resulting estimates exploratory. *MEAK7* demonstrated an AUC of 0.795 (95% CI: 0.719–0.871), with a sensitivity of 63.8% and a specificity of 85.3% at a cut-off of ≥4.279. The AUCs for the selected driver genes ranged from 0.579 to 0.751 (Table 1). These results suggest that *MEAK7* expression may facilitate tissue-level discrimination between PDAC tumor and adjacent non-tumor samples in this cohort; however, they should not be construed as indicative of clinical diagnostic performance.

### 2.3. Descriptive Proteomic Analysis of MEAK7 in Relation to Clinical and Demographic Characteristics Using the UALCAN Database

**Figure 2:**
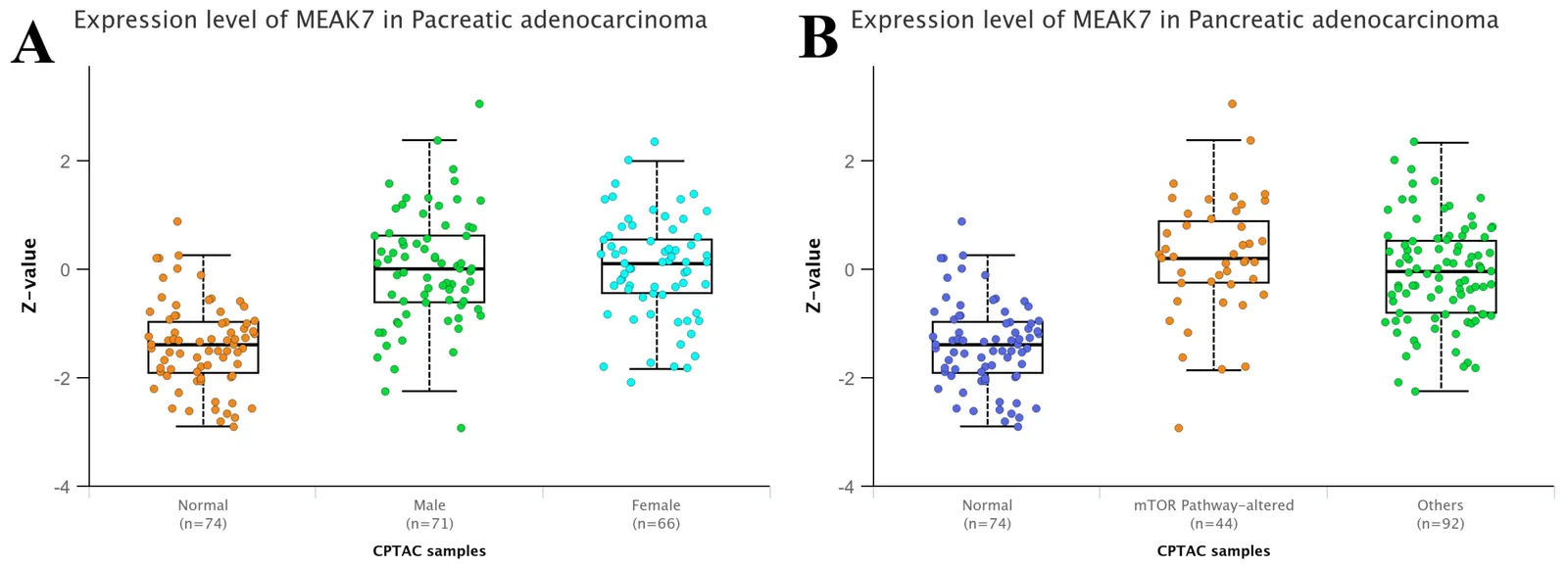
Distribution of MEAK7 protein expression in pancreatic adenocarcinoma derived from CPTAC data according to (**A**) gender and (**B**) mTOR pathway-altered/unaltered.

UALCAN-provided pairwise comparisons showed significantly higher MEAK7 protein expression in male and female PDAC samples than in normal pancreatic tissue (adj*p* = 3.36 × 10^−15^ and adj*p* = 1.76 × 10^−16^, respectively). Within the tumor cohort, no statistically significant difference was detected between male and female patients (adj*p* = 0.916) (Figure 2A).

Similarly, MEAK7 protein expression was significantly higher in both mTOR pathway–altered and other PDAC samples than in normal pancreatic tissue (adj*p* = 3.10 × 10^−12^ and adj*p* = 3.45 × 10^−18^, respectively). However, no statistically significant difference was detected between mTOR pathway–altered and other tumor samples (adj*p* = 0.126) (Figure 2B). These platform-generated comparisons were interpreted as exploratory and were not considered evidence of subgroup-specific equivalence or differential *MEAK7* expression.

### 2.4. Comparison of MEAK7 Expression Levels in Normal, Tumor, and Metastatic Tissues

For comparisons involving normal, primary tumor, and metastatic groups, an omnibus Kruskal–Wallis test was followed by Dunn’s post hoc multiple-comparison procedure as implemented by TNMplot. No additional Benjamini–Hochberg correction was applied to these platform-generated pairwise comparisons.

**Figure 3:**
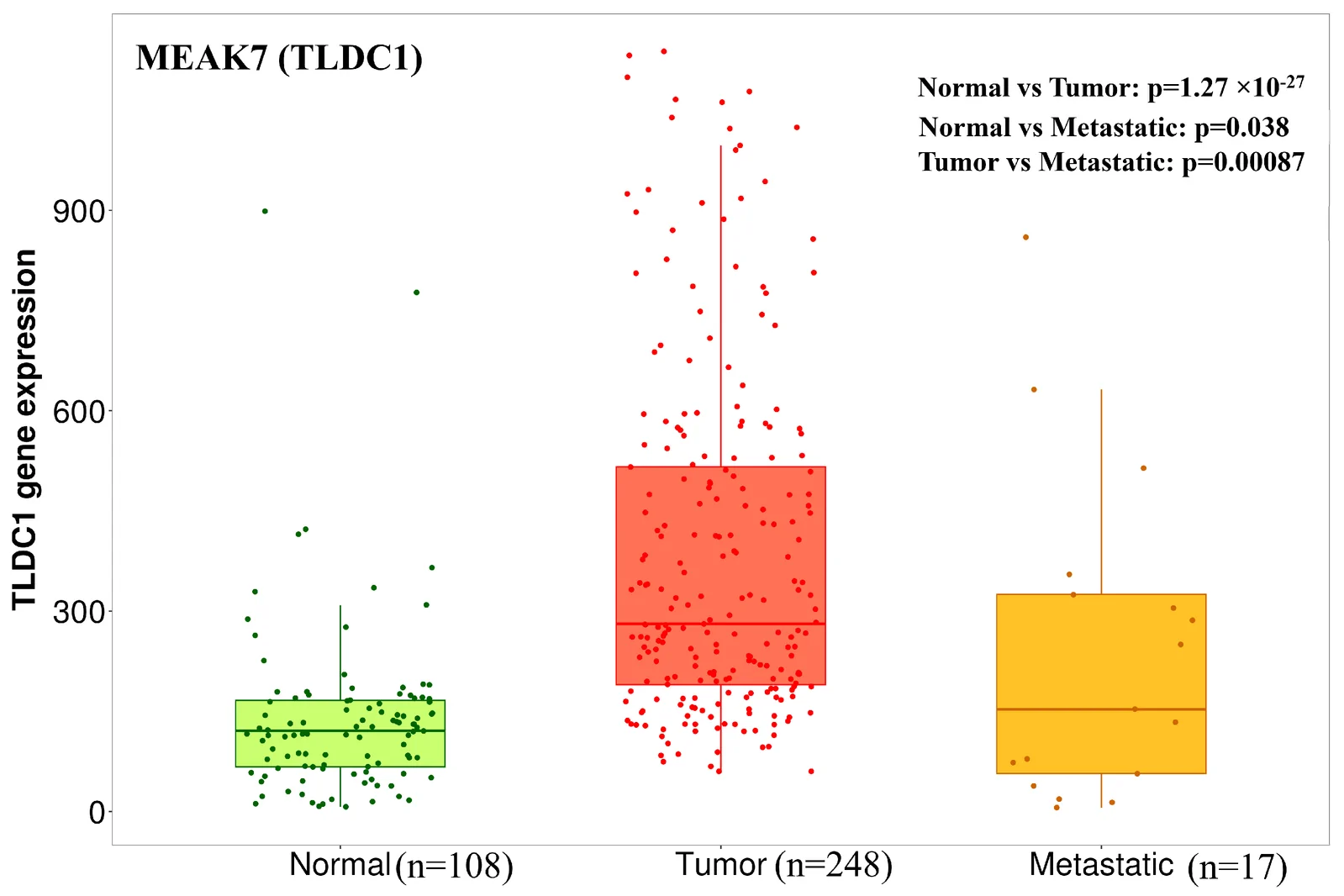
Comparative analysis of *MEAK7* gene expression across normal, primary tumor, and metastatic tissues in pancreatic cancer.

Analysis of *MEAK7* expression levels in normal pancreatic tissue, primary tumor tissue, and metastatic tissue within the PAAD cohort revealed that *MEAK7* expression was significantly upregulated in primary tumor tissue compared with normal pancreatic tissue (*p* = 1.27 × 10^−27^). In metastatic tissue, *MEAK7* expression was significantly upregulated relative to that in normal pancreatic tissue (*p* = 0.038) but significantly downregulated compared to that in primary tumor tissues (*p* = 0.00087) (Figure 3). Despite the metastatic cohort in the TNMplot database consisting of only 17 samples, statistical analyses were conducted using the Kruskal–Wallis test, followed by Dunn’s post hoc multiple-comparison test. This approach is suitable for non-parametric comparisons with unequal group sizes. However, due to the limited sample size, the metastatic findings should be interpreted with caution. Notably, this metastatic pattern was not reproduced in GSE71729. Whereas TNMplot showed lower *MEAK7* expression in metastatic than in primary tumor tissues, GSE71729 showed the opposite direction, with significantly higher expression in metastatic samples. Therefore, the relationship between *MEAK7* expression and metastatic progression was considered inconsistent across datasets and should not be interpreted as evidence of a uniform metastatic expression trajectory.

### 2.5. Co-Expression Analysis Identifies the Top 20 Genes Positively Correlated with MEAK7 Expression

In the Spearman correlation analysis performed on PAAD tumor tissues, robust and highly statistically significant positive correlations were identified between *MEAK7* expression and the expression of several genes. The strongest correlation was observed with *CTTN* (Spearman’s *ρ* = 0.74; *p* = 3.89 × 10^−41^), followed by *STAU1* (*ρ* = 0.73; *p* = 5.25 × 10^−39^), *SMARCA4* (*ρ* = 0.72; *p* = 3.6 × 10^−37^), and *SFN* (*ρ* = 0.72; *p* = 7.39 × 10^−38^). Furthermore, the genes *GSS* (*ρ* = 0.71; *p* = 6.17 × 10^−36^), *EFNA5* (*ρ* = 0.71; *p* = 6.17 × 10^−36^), *OPA1* (*ρ* = 0.71; *p* = 1.0 × 10^−35^), *SFXN1* (*ρ* = 0.71; *p* = 5.51 × 10^−36^), and *CDCP1* (*ρ* = 0.71; *p* = 1.19 × 10^−35^) also demonstrated a high degree of positive correlation with *MEAK7.* Additional genes exhibiting a moderate-to-high level of correlation included *ARFGEF1* (*ρ* = 0.70; *p* = 3.91 × 10^−35^), *SRD5A1* (*ρ* = 0.70; *p* = 6.11 × 10^−35^), *KDM2A* (*ρ* = 0.70; *p* = 1.57 × 10^−34^), *KBTBD2* (*ρ* = 0.70; *p* = 4.96 × 10^−35^), *CARS* (*ρ* = 0.70; *p* = 1.61 × 10^−35^), and *PALB2* (*ρ* = 0.70; *p* = 3.02 × 10^−35^). Additionally, the genes *BRD8* (*ρ* = 0.69; *p* = 9.59 × 10^−34^), *MTIF2* (*ρ* = 0.69; *p* = 2.53 × 10^−33^), *TUFT1* (*ρ* = 0.69; *p* = 1.33 × 10^−33^), *STK24* (*ρ* = 0.69; *p* = 2.53 × 10^−33^), and *KCMF1* (*ρ* = 0.69; *p* = 3.7 × 10^−33^) were also significantly and positively associated with *MEAK7* expression (Table 2). Additionally, the adjusted *p*-values after FDR correction are shown in Table 2.

### 2.6. Prognostic Significance of MEAK7 Expression in PAAD

**Figure 4:**
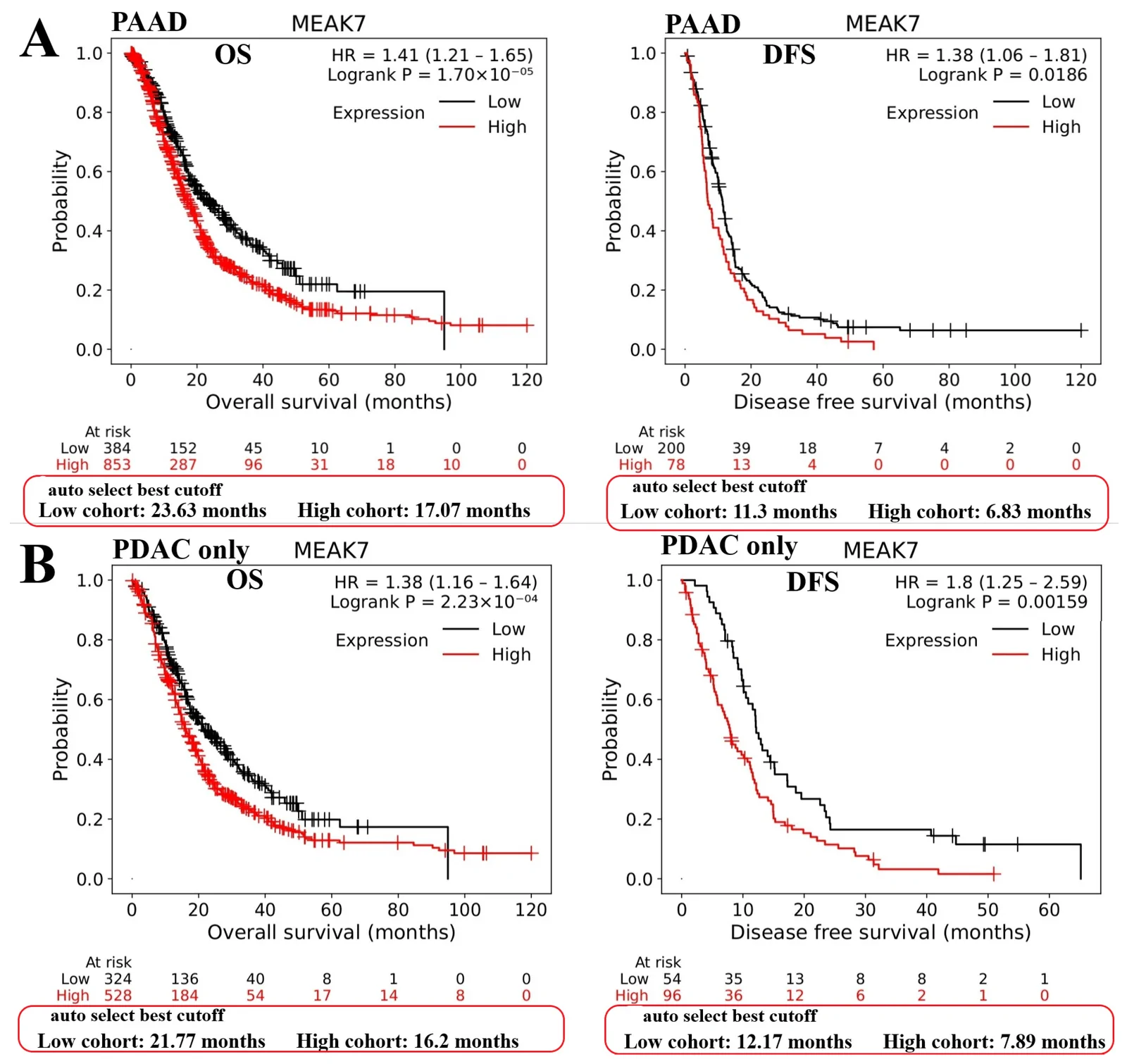
Prognostic significance of *MEAK7* expression in pancreatic cancer. Kaplan–Meier survival analyses based on *MEAK7* mRNA expression levels are shown. (**A**) OS and DFS in PAAD; (**B**) OS and DFS in PDAC-only cases.

In the GEO database cohort (*n* = 1237) [16] elevated *MEAK7* expression was correlated with reduced overall survival (HR = 1.41, 95% CI: 1.21–1.65, nominal log-rank *p* = 1.70 × 10^−5^, FDR-adjusted *p* = 3.68 × 10^−4^; cutoff: 209); the median overall survival (OS) was 23.63 months in the low-expression group compared to 17.07 months in the high-expression group. In the disease-free survival (DFS) analysis (*n* = 278), patients with high *MEAK7* expression showed shorter DFS (HR = 1.38, 95% CI: 1.06–1.81, nominal log-rank *p* = 0.0186, FDR-adjusted *p* = 0.352; cutoff: 768), with median DFS durations of 11.30 months in the low-expression group and 6.83 months in the high-expression group. However, this association did not remain statistically significant after correction. In analyses restricted to PDAC cases (*n* = 852), high *MEAK7* expression was similarly associated with decreased overall survival (HR = 1.38, 95% CI: 1.16–1.64, nominal log-rank *p* = 2.23 × 10^−4^, FDR-adjusted *p* = 0.00424; cutoff: 211); the median OS was 21.77 months in the low-expression group and 16.2 months in the high-expression group. Similarly, in the PDAC disease-free survival analysis (*n* = 150), high *MEAK7* expression was associated with shorter DFS (HR = 1.80, 95% CI: 1.25–2.59, log-rank nominal log-rank *p* = 0.00159, FDR-adjusted *p* = 0.0222; cutoff: 122), with median DFS durations of 12.17 and 7.89 months in the low- and high-expression groups, respectively (Figure 4).

### 2.7. The Relationship of MEAK7 Expression with Survival, Age, and Stage (DoSurvive Analysis)

The association between *MEAK7* expression and survival outcomes in patients with PAAD (*n* = 174) was assessed using Kaplan–Meier analysis in the DoSurvive database. Patients were categorized into high- (*n* = 87) and low-expression (*n* = 87) groups based on their *MEAK7* expression levels. Multivariate Cox regression analysis for overall survival (OS) in PAAD indicated that elevated *MEAK7* expression served as an independent adverse prognostic factor (HR = 1.51, 95% CI: 1.23–1.85, adj*p* = 7.02 × 10^−3^). Furthermore, age at diagnosis was independently correlated with diminished OS (HR = 1.03, 95% CI: 1.01–1.05; *p* = 0.0068). Conversely, sex (*p* = 0.371) and tumor stage (*p* = 0.572) did not exhibit significant independent associations with OS in the adjusted model. Age at diagnosis was independently linked to overall survival, with each additional year of age corresponding to an approximate 3% increase in mortality risk.

Multivariate Cox regression analysis of the Disease-free Interval (DFI) in PAAD identified elevated *MEAK7* expression as an independent adverse prognostic factor (HR = 2.26, 95% CI: 1.44–3.54, adj*p* = 4.31 × 10^−2^). Age at diagnosis did not show a significant independent association with the DFI (*p* = 0.53). Additionally, tumor stage was not correlated with DFI in the adjusted model (*p* = 0.996). Conversely, male sex was significantly correlated with a reduced risk of a DFI event compared to female sex (HR = 0.39, 95% CI: 0.16–0.99; *p* = 0.048). The DoSurvive platform does not offer proportional hazards testing or model diagnostic outputs.

### 2.8. Immune Infiltration Analysis Results

Analyses examining the correlation between *MEAK7* and immune cell infiltration were performed using the statistical frameworks incorporated in the TIMER 3.0 platform. Spearman correlation coefficients and their corresponding significance values were derived directly from the validated analytical pipeline. To address multiple comparisons in the immune infiltration analyses, the Benjamini–Hochberg false discovery rate (FDR) correction was applied.

In the PAAD cohort, no significant correlation was observed between *MEAK7* expression and tumor purity. *MEAK7* expression was positively correlated with B-cell abundance (TIMER) (*ρ* = 0.262, nominal *p* = 5.69 × 10^−4^, FDR = 0.00142), CD8^+^ T-cell abundance (*ρ* = 0.217, nominal *p* = 4.51 × 10^−3^, FDR = 0.00667), and neutrophil abundance (*ρ* = 0.216, nominal *p* = 4.67 × 10^−3^, FDR = 0.00667). In contrast, *MEAK7* expression was negatively correlated with CD4^+^ T-cell abundance (*ρ* = −0.190, nominal *p* = 1.33 × 10^−2^, FDR = 0.01663).

Among myeloid cell subsets, *MEAK7* expression was negatively correlated with monocyte abundance (QUANTISEQ) (*ρ* = −0.294, nominal *p* = 1.02 × 10^−4^, FDR = 0.00102) and positively correlated with M0 macrophages (CIBERSORT) (*ρ* = 0.234, nominal *p* = 2.16 × 10^−3^, FDR = 0.00432). Regarding stromal and immune-related components, *MEAK7* expression was positively correlated with cancer-associated fibroblasts (EPIC) (*ρ* = 0.279, nominal *p* = 2.25 × 10^−4^, FDR = 0.00113), eosinophils (CIBERSORT) (*ρ* = 0.158, nominal *p* = 3.91 × 10^−2^, FDR = 0.04200), and CD274 (PD-L1) estimated by the TIDE algorithm (*ρ* = 0.262, nominal *p* = 5.45 × 10^−4^, FDR = 0.00142). *MEAK7* expression also showed a weak negative correlation with NK cells (QUANTISEQ) (*ρ* = −0.156, nominal *p* = 4.20 × 10^−2^, FDR = 0.04200). No significant association was observed with activated mast cells, γδ T cells, or regulatory T cells (Tregs) (Table 3).

Among patients with high *MEAK7* expression, none of the evaluated immune or stromal components showed a statistically significant association with OS after correction for multiple comparisons. CD4^+^ T-cell infiltration estimated using TIMER showed the lowest nominal *p* value. Patients with high *MEAK7* expression and high CD4^+^ T-cell infiltration (Group 4) had a lower estimated mortality hazard than patients with high *MEAK7* expression and low CD4^+^ T-cell infiltration (Group 3; HR = 0.541, nominal *p* = 0.0442). However, this association did not remain statistically significant after Benjamini–Hochberg correction (FDR-adjusted *p* = 0.4420). No nominally significant OS differences were observed for cancer-associated fibroblasts, CD8^+^ T cells, CD274/PD-L1, eosinophils, M1 macrophages, monocytes, NK cells, neutrophils, or B cells.

Similarly, none of the evaluated immune or stromal components was significantly associated with DSS. The lowest nominal *p* values were observed for CD4^+^ T cells (HR = 0.552, nominal *p* = 0.0519) and eosinophils (HR = 0.568, nominal *p* = 0.0619); both yielded an FDR-adjusted *p* value of 0.3095. Overall, immune/stromal infiltration did not significantly stratify OS or DSS among patients with high *MEAK7* expression after correction for multiple testing (Table 4).

### 2.9. STRING Database-Based Functional Association Analysis of MEAK7

**Figure 5:**
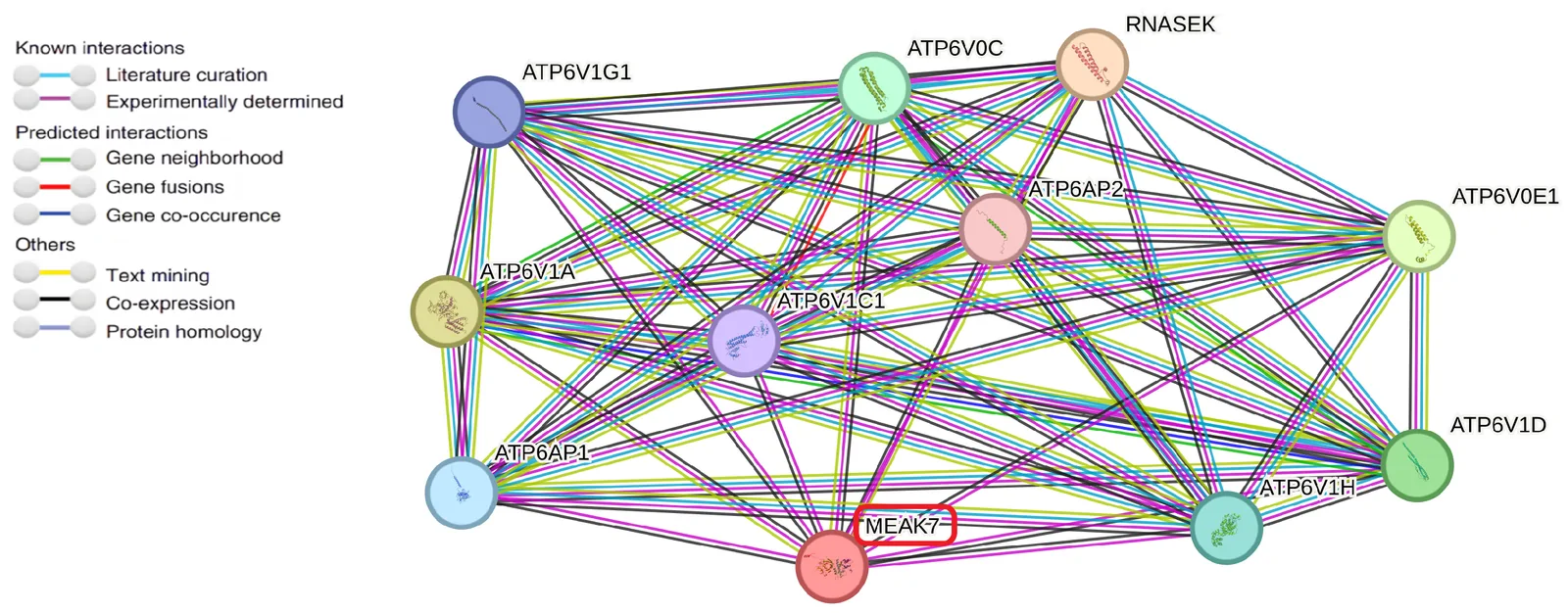
STRING-derived protein functional association network involving MEAK7 (highlighted by a red frame) and various subunits of the vacuolar H+-ATPase (V-ATPase) complex, including ATP6V1A, ATP6V1G1, ATP6V1C1, ATP6AP1, ATP6AP2, ATP6V0C, ATP6V0E1, ATP6V1D, ATP6V1H, and RNASEK. The displayed edges represent functional associations inferred by STRING and should not be interpreted as direct physical protein–protein interactions.

STRING analysis identified functional associations between MEAK7 and several V-ATPase-related proteins, including ATP6V1A, ATP6V1C1, ATP6V1D, ATP6V1H, ATP6V0C, ATP6V0E1, ATP6AP1, ATP6AP2, RNASEK, and ATP6V1G1. The functional association network was supported by curated literature and experimental evidence, as well as predicted relationships based on co-expression, gene co-occurrence, gene neighborhood, gene fusion, text mining, and protein homology analysis. Within the STRING-derived functional association network, MEAK7 was connected with multiple V-ATPase-related proteins. These findings suggest a potential functional relationship but do not demonstrate direct physical interactions or a central regulatory role (Figure 5). The combined score results of the gene–gene interactions are presented in Table 5. No additional multiple-testing correction was applicable to the STRING network analysis because associations were selected using the predefined STRING confidence-score threshold rather than hypothesis-test *p* values.

### 2.10. Prediction of MEAK7 miRNA Interactions Using TargetScan 8.0 and miRDB

**Figure 6:**
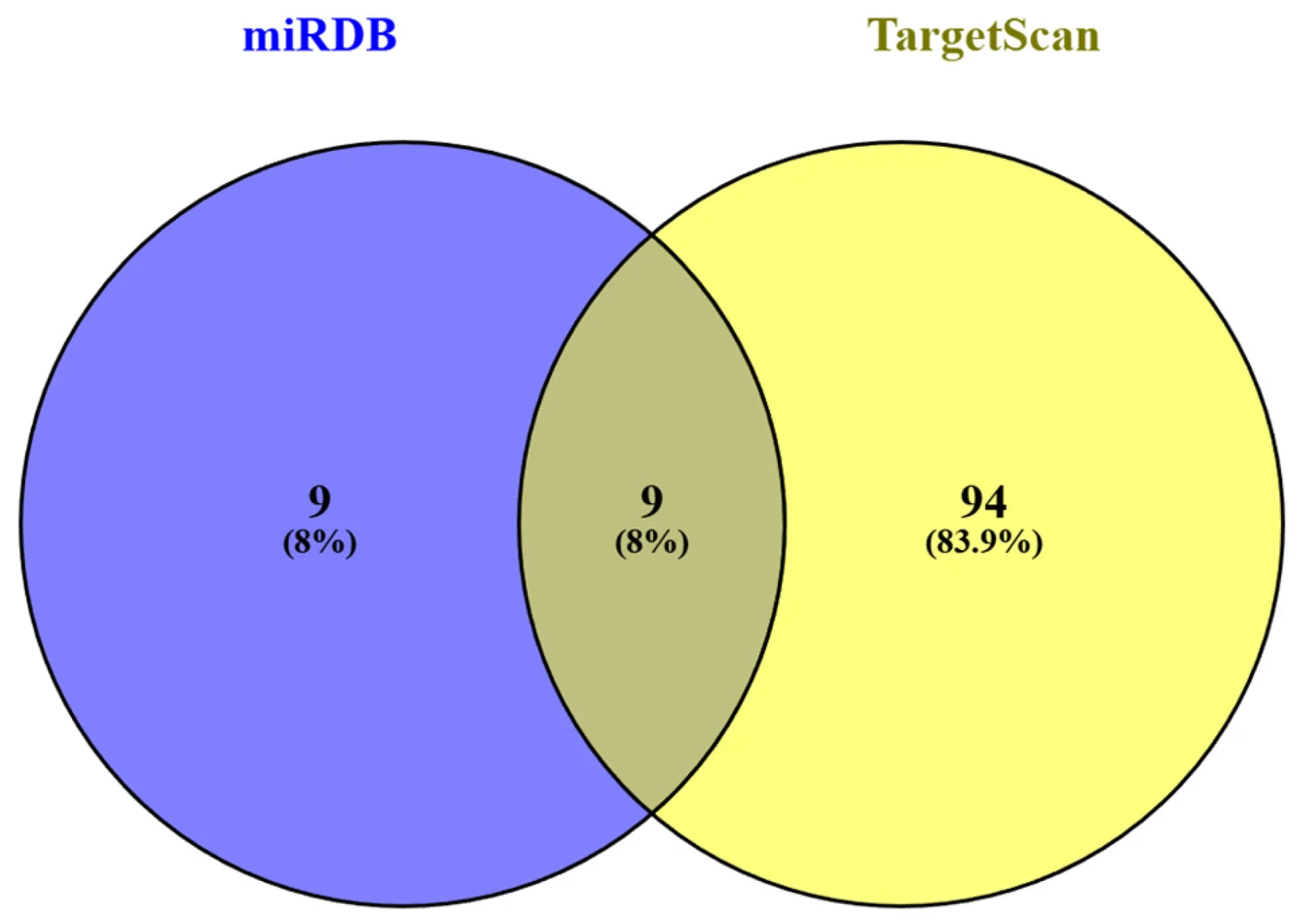
miRNA–target prediction overlap analysis for MEAK7.

MicroRNA (miRNA) target prediction analysis for MEAK7 was conducted using the miRDB and TargetScan databases. Comparative analysis indicated that nine miRNAs were consistently predicted by both platforms. In addition, nine miRNAs were uniquely identified by miRDB, whereas 94 miRNAs were exclusively predicted by TargetScan. The miRNAs predicted by both databases were hsa-miR-582-5p, hsa-miR-135b-5p, hsa-miR-135a-5p, hsa-miR-202-3p, hsa-miR-432-5p, hsa-miR-6855-3p, hsa-miR-4513, hsa-miR-3670, and hsa-miR-149-3p. The shared miRNAs were selected for further analysis (Figure 6 and Table 6).

### 2.11. LncRNAs Associated with PAAD Identified Through the LncRNADisease Database

**Figure 7:**
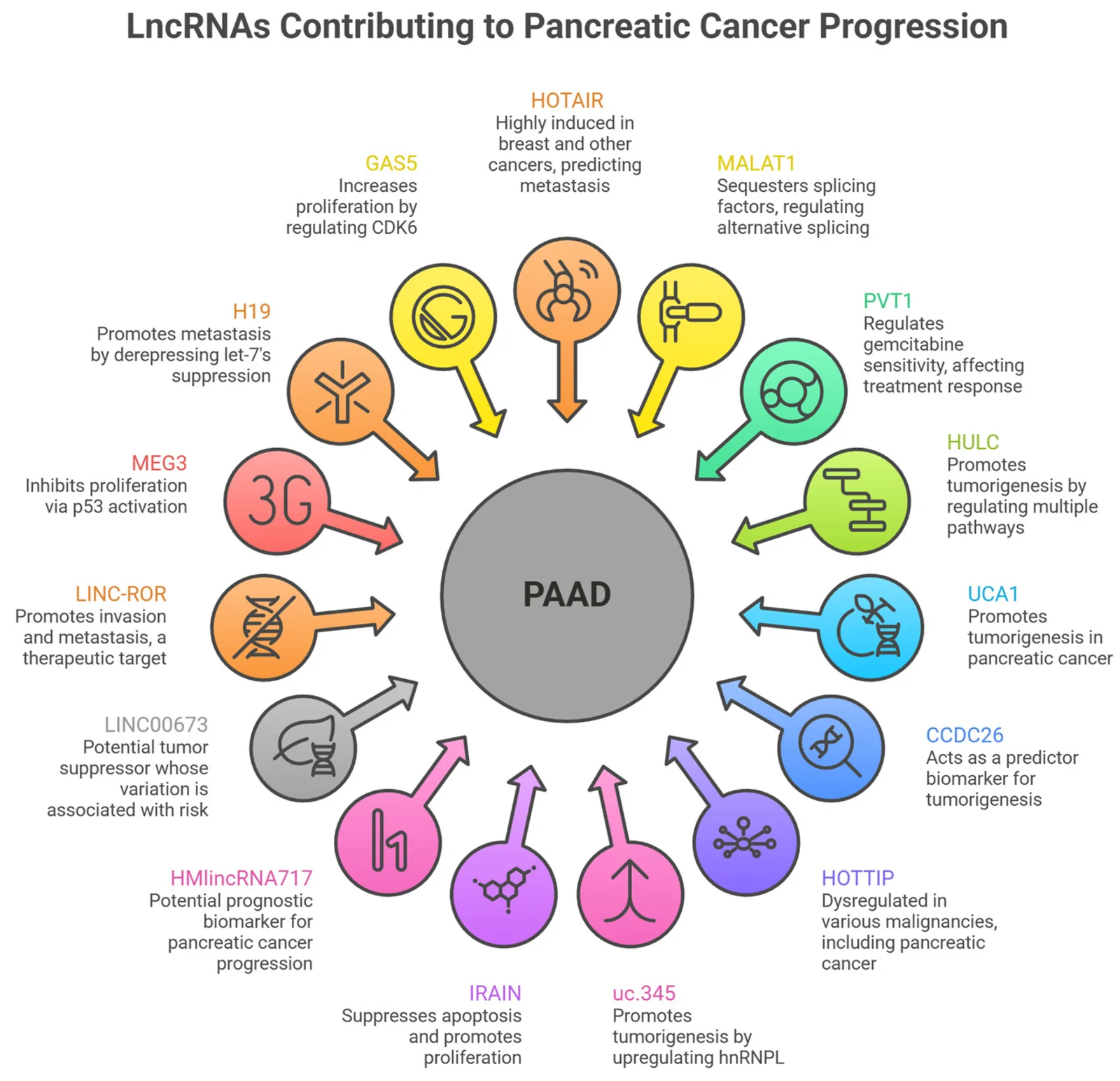
This figure provides a summary of the long non-coding RNAs (lncRNAs) that have been reported in the literature as being associated with the progression of pancreatic cancer, along with the primary functional characteristics of these molecules.

The roles of long non-coding RNAs (lncRNAs) in processes such as proliferation, invasion, metastasis, apoptosis, alternative splicing, chemotherapy response, and tumorigenesis in pancreatic cancer are depicted in Figure 7. Each lncRNA is strategically placed around a central structure symbolizing a pancreatic cancer cell in accordance with its functional association, as documented in the literature. Figure 7 visually illustrates the functional diversity and scope of lncRNAs associated with pancreatic cancer.

### 2.12. ENCORI-Based Expression Correlation Analysis of MEAK7 with Candidate miRNAs and lncRNAs

Analyses of the correlation between MEAK7 and candidate miRNAs, as well as lncRNAs, were conducted utilizing the statistical frameworks integrated within the ENCORI platform. The correlation coefficients and their associated significance values were directly derived from these validated analytical pipelines. Because adjusted *p* values were not available from the platform output for these targeted ENCORI correlation analyses, the reported *p* values are nominal and were not subjected to additional multiple-testing correction. Accordingly, these results were treated as exploratory and hypothesis-generating.

#### 2.12.1. miRNAs vs. *MEAK7 (TLDC1)* Correlations

**Figure 8:**
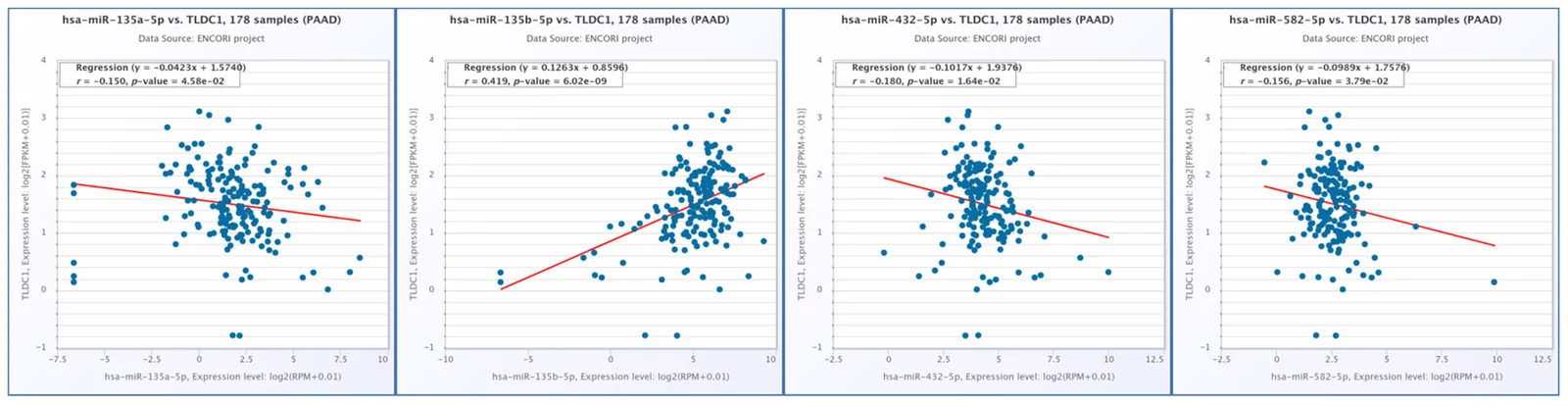
Correlation analyses were conducted to examine the association between *TLDC1* expression and selected microRNAs (miRNAs) in pancreatic adenocarcinoma (PAAD) samples.

In the PAAD cohort (*n* = 178), correlation analyses using the ENCORI database revealed various associations between *MEAK7* (*TLDC1*) and miRNA expressions. A statistically significant positive correlation was observed between hsa-miR-135b-5p and *MEAK7* (*TLDC1*) expression (*r* = 0.419, *p* = 6.02 × 10^−9^). Conversely, statistically significant negative correlations were identified between *MEAK7* (*TLDC1*) and hsa-miR-135a-5p, hsa-miR-432-5p, and hsa-miR-582-5p (*r* = −0.150, *p* = 0.0458; *r* = −0.180, *p* = 0.0164; and *r*= −0.156, *p* = 0.0379, respectively) (Figure 8). No statistically significant correlations were found between *TLDC1* expression and the other miRNAs (hsa-miR-149-3p, hsa-miR-202-3p, hsa-miR-3670, hsa-miR-4513, and hsa-miR-6855-3p) (*p* > 0.05).

#### 2.12.2. LncRNAs vs. *MEAK7 (TLDC1)* Correlations

**Figure 9:**
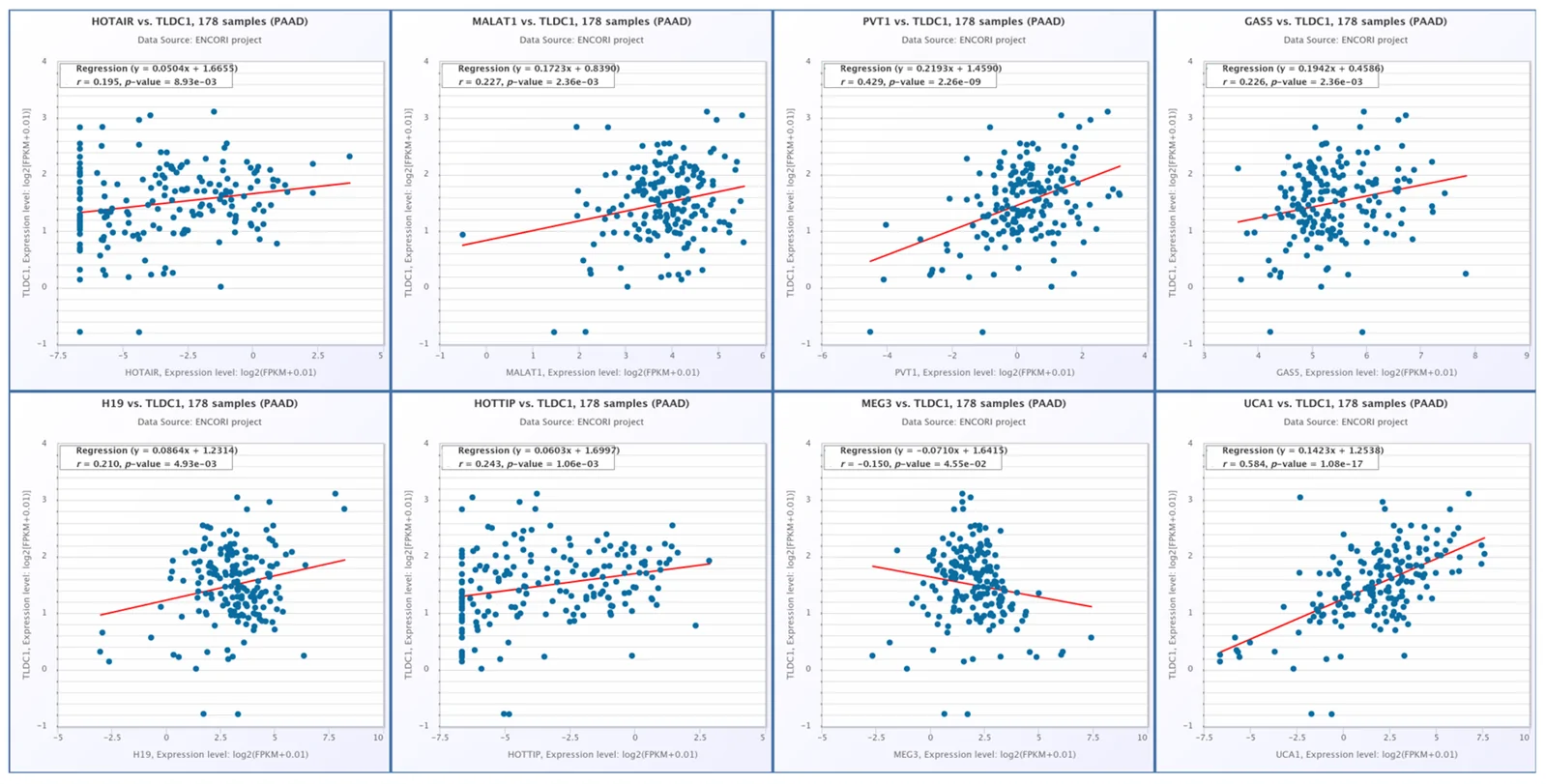
Correlation analyses were conducted to examine the association between *TLDC1* expression and selected long non-coding RNAs (lncRNAs) in pancreatic adenocarcinoma (PAAD) samples.

In the correlation analyses conducted within the PAAD cohort (*n* = 178) using the ENCORI database, statistically significant associations were identified between MEAK7 (TLDC1) expression and specific lncRNA expression levels. A moderate positive correlation was observed between *UCA1* and *MEAK7* (*TLDC1*) (*r* = 0.584, *p* = 1.08 × 10^−17^). Additionally, positive correlations were noted between *TLDC1* and *PVT1* (*r* = 0.429, *p* = 2.26 × 10^−9^), *HOTTIP* (*r* = 0.243, *p* = 1.06 × 10^−3^), *GAS5* (*r* = 0.226, *p* = 2.36 × 10^−3^), *H19* (*r* = 0.210, *p* = 4.93 × 10^−3^), *MALAT1* (*r* = 0.227, *p* = 2.36 × 10^−3^), and *HOTAIR* (*r* = 0.195, *p* = 8.93 × 10^−3^). Conversely, a negative correlation was observed between *MEG3* and *MEAK7* (*TLDC1*) (*r* = −0.150, *p* = 4.55 × 10^−2^) (Figure 9). No statistically significant association was observed between *MEAK7 (TLDC1)* expression and *LINC-ROR* or *CCDC26* expression (*p* > 0.05).

### 2.13. External Assessment of MEAK7 Expression in Early-Stage PDAC Using GSE62165

**Figure 10:**
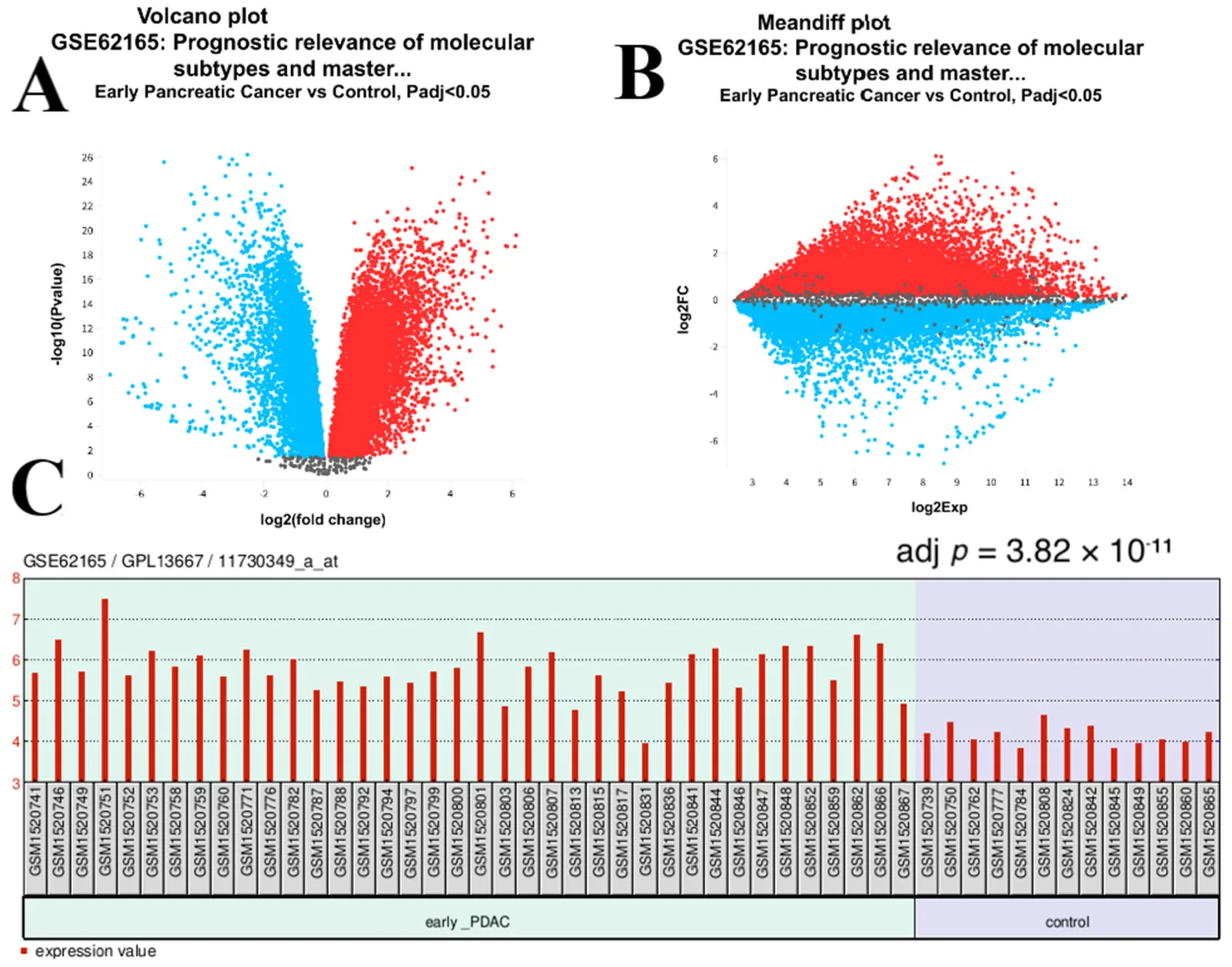
Differential gene expression analyses were conducted between early-stage pancreatic cancer and control samples using the GSE62165 dataset. (**A**) Volcano plot; (**B**) mean-difference plot; (**C**) expression of probe 11730349_a_at in early-stage PDAC and control tissues. In panels (**A**,**B**), red and blue indicate significantly upregulated and downregulated probes, respectively, whereas gray indicates nonsignificant probes.

In the GSE62165 cohort, which included 38 early-stage PDAC tissues and 13 non-tumor pancreatic control tissues, GEO2R/limma analysis showed higher *MEAK7* expression in the PDAC group. Probe 11730349_a_at, selected as the representative KIAA1609 probe based on the lowest adjusted *p* value, showed a log2FC of 1.604 (nominal *p* = 2.39 × 10^−12^; Benjamini–Hochberg FDR-adjusted *p* = 3.82 × 10^−11^) (Figure 10A–C). Thus, the increased *MEAK7* expression remained statistically significant after correction for multiple testing.

### 2.14. External Assessment of MEAK7 Expression in GSE183795

**Figure 11:**
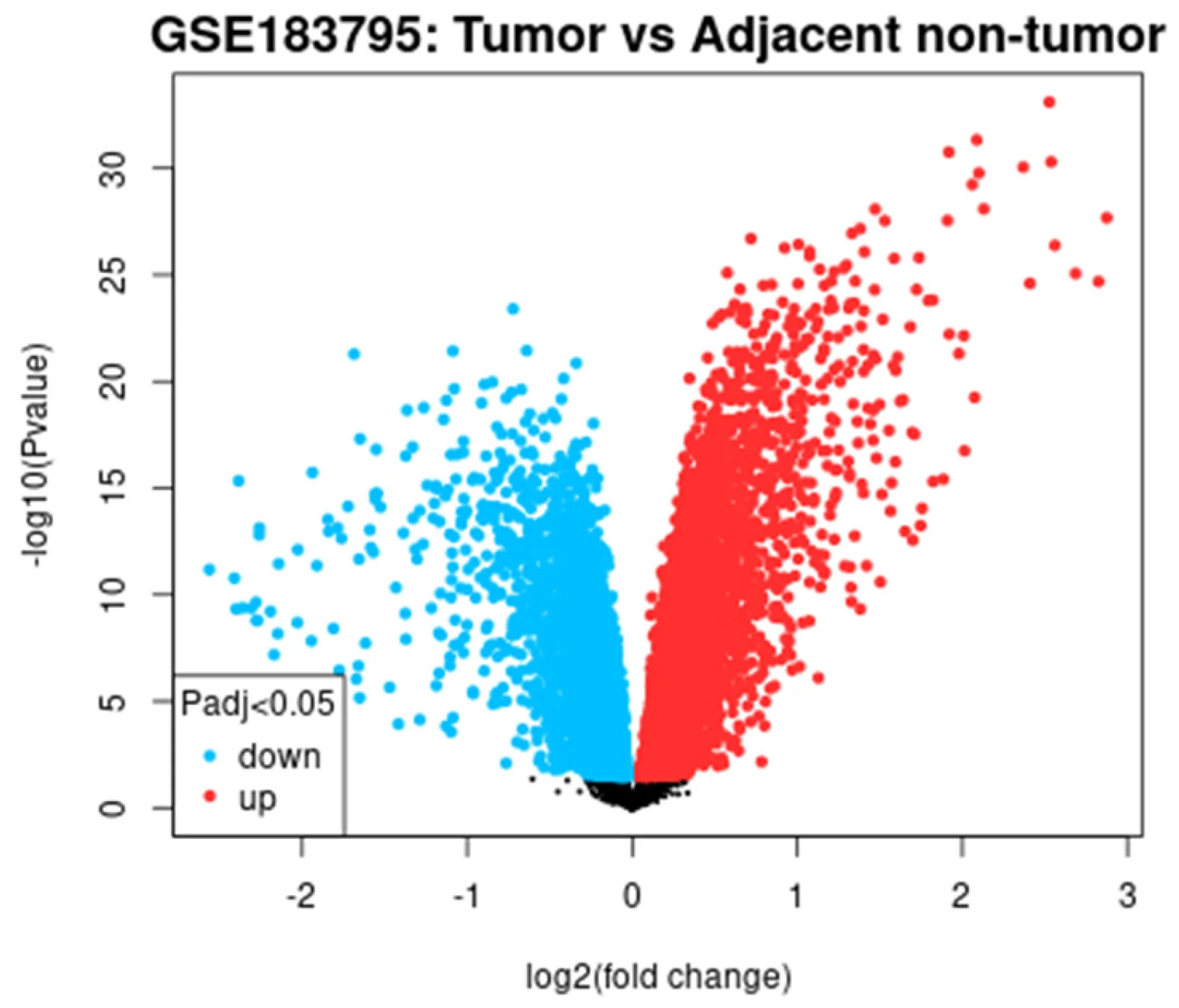
Volcano plot of differential gene expression between PDAC tumor and adjacent non-tumor pancreatic tissues in the GSE183795 cohort. Red and blue dots indicate significantly upregulated and downregulated probes, respectively, whereas black dots indicate nonsignificant probes (FDR-adjusted *p* ≥ 0.05).

In GSE183795, comprising 139 PDAC tumor tissues and 102 adjacent non-tumor pancreatic tissues, *MEAK7* expression was significantly higher in tumor tissues. Differential-expression analysis using GEO2R/limma showed that this difference remained statistically significant after Benjamini–Hochberg correction (FDR-adjusted *p* = 9.25 × 10^−14^) (Figure 11). These findings provide external supportive evidence for increased *MEAK7* expression in PDAC but were not interpreted as independent clinical validation.

### 2.15. Dataset-Dependent MEAK7 Expression in Primary and Metastatic PDAC: GSE71729

**Figure 12:**
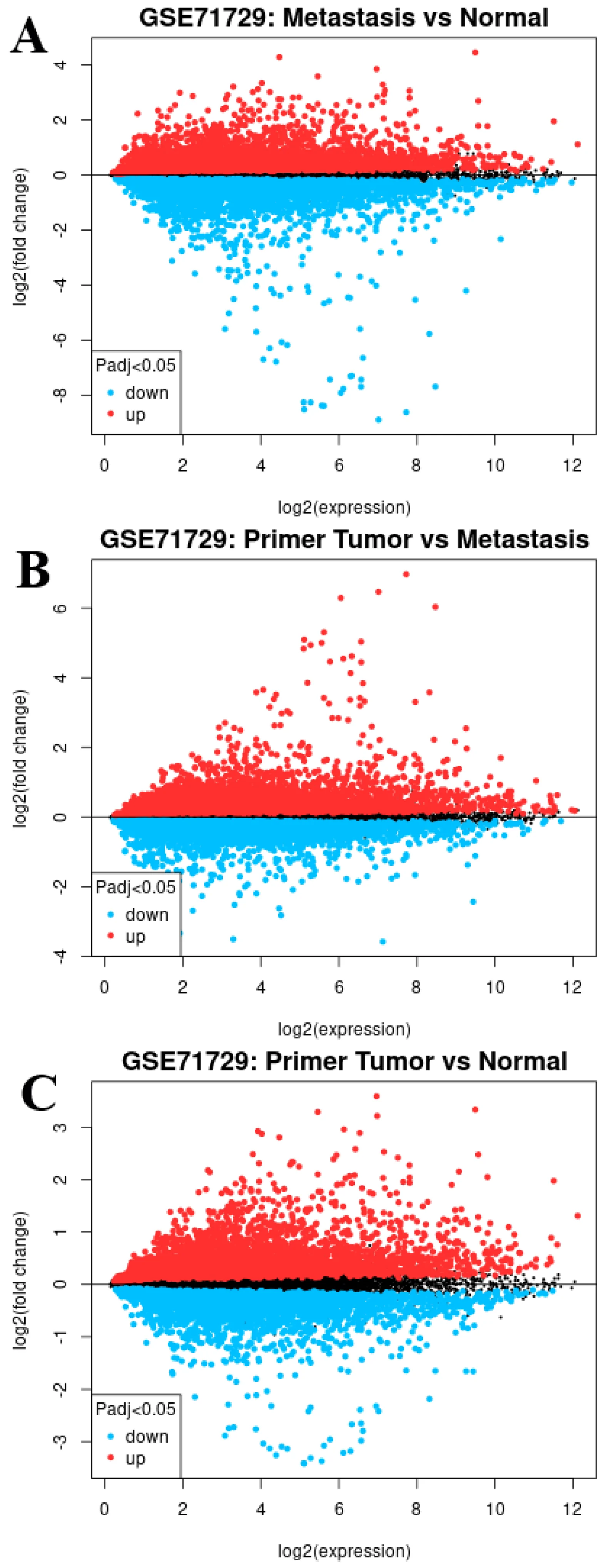
Differential gene expression analyses were performed between (**A**) metastatic tissues and normal pancreatic tissues; (**B**) primary tumor and metastatic tissues; and (**C**) primary tumor and normal pancreatic tissue using the GSE71729 data set. Red and blue dots indicate significantly upregulated and downregulated probes, respectively, whereas black dots indicate nonsignificant probes (FDR-adjusted *p* ≥ 0.05).

Analysis of GSE71729 demonstrated significant differences in *MEAK7* expression among normal pancreatic, primary PDAC, and metastatic tissue groups. *MEAK7* expression was higher in primary tumors than in normal pancreatic tissues (logFC = 0.431; FDR-adjusted *p* = 3.00 × 10^−6^). In the primary tumor-versus-metastasis contrast, the negative logFC (−0.356; FDR-adjusted *p* = 1.02 × 10^−5^) indicated higher *MEAK7* expression in metastatic tissues than in primary tumors. *MEAK7* expression was also higher in metastatic than in normal pancreatic tissues (logFC = 0.790; FDR-adjusted *p* = 7.13 × 10^−14^) (Figure 12). Importantly, the direction of the primary-versus-metastatic difference in GSE71729 was opposite to that observed in TNMplot, where *MEAK7* expression was lower in metastatic than in primary tumor tissues. Thus, the available datasets do not support a consistent direction of *MEAK7* expression change during metastatic progression.

### 2.16. External Transcriptomic Assessment of MEAK7 Expression Using the EMBL-EBI Expression Atlas

*MEAK7* expression was externally assessed at the transcriptomic level using the EMBL-EBI Expression Atlas. In pancreatic cancer cell line datasets, *MEAK7* showed detectable expression in multiple pancreatic cancer cell lines (Table 7).

## 3. Discussion

This study presents a comprehensive multi-platform bioinformatic evaluation that incorporates transcriptomic, proteomic, clinical, immune-related, and regulatory analyses of MEAK7 in pancreatic ductal adenocarcinoma (PDAC). The main findings indicate that *MEAK7* is consistently upregulated at both the transcriptomic and proteomic levels in PDAC, including in early-stage disease, and that high *MEAK7* expression is associated with adverse survival outcomes. Importantly, multivariate Cox regression analyses revealed that, after adjustment for age, sex, and tumor stage, *MEAK7* was an independent predictor of both overall survival and disease-free interval. These findings suggest that *MEAK7* may have clinical significance as a prognostic biomarker candidate in PDAC and may reflect biologically meaningful alterations associated with pancreatic tumor progression.

Previous experimental evidence supports the current findings. In vitro studies have shown that MEAK7 may contribute to pancreatic adenocarcinoma cell proliferation [17]. Consistent with this observation, our analyses demonstrated detectable *MEAK7* expression across multiple pancreatic cancer cell-line datasets, while patient-derived PDAC samples, including early-stage tumors, showed elevated MEAK7 expression relative to the corresponding control tissues.

Moreover, the association of high *MEAK7* expression with adverse survival outcomes and its independent prognostic value in multivariate Cox models extend the significance of MEAK7 from cell-based observations to clinically defined patient cohorts. Thus, the present study complements previous experimental data by providing a broader transcriptomic, proteomic, prognostic, and regulatory framework for evaluating *MEAK7* in PDAC.

In pancreatic cancer, *KRAS* and *TP53* mutations are considered key oncogenic driver genes that play critical roles in tumor initiation and progression, profoundly affecting cell growth, cellular stress response, and genomic stability [18,19]. The observation of higher *MEAK7* expression levels in *KRAS* and *TP53* mutant tumors in our PAAD cohort suggests that *MEAK7* upregulation may be associated with major oncogenic and tumor-suppressor pathway alterations in pancreatic cancer.

*KRAS* mutations are closely associated with the activation of the PI3K/AKT/mTOR signaling pathway [20,21]. While MEAK7 has been associated with mTOR signaling [9,10], the current CPTAC analysis did not reveal a significant difference in MEAK7 protein expression between mTOR pathway–altered and non-altered PDAC samples. Consequently, our results do not indicate a direct correlation between *MEAK7* expression levels and mTOR pathway alteration status. Rather, *MEAK7* overexpression seems to be a general feature of PDAC tissues, regardless of mTOR pathway classification. Nonetheless, functional experimental studies are necessary to clarify the causal mechanisms underlying this relationship.

To contextualize the tissue expression findings, *MEAK7* was analyzed in conjunction with selected PDAC driver genes through ROC analysis. Within the GSE62452 cohort, *MEAK7* demonstrated an AUC of 0.795, while the AUCs for the selected driver genes varied from 0.579 to 0.751. These results indicate that *MEAK7* expression may signify a tumor-associated expression difference in this cohort; however, they do not confirm clinical diagnostic utility. Given that the gene-specific cut-offs and performance estimates were both derived and assessed within the same cohort, and considering that adjacent non-tumor tissues do not serve as an independent healthy control group, these estimates may be specific to the cohort and platform. Therefore, validation of a predetermined cut-off in independent, well-characterized cohorts, ideally employing additional analytical platforms and prospective samples, is necessary before any diagnostic application of *MEAK7* can be considered.

Survival analyses demonstrated that elevated *MEAK7* expression in PDAC was associated with unfavorable overall survival (OS) and disease-free survival (DFS). In the multivariable Cox analyses, this association persisted after adjustment for age, sex, and tumor stage. However, these findings should not be interpreted as establishing MEAK7 as an independently validated prognostic biomarker, because the analyses were retrospective and were limited to the covariates and statistical framework available within the analyzed datasets. Given the limited ability of conventional clinicopathological parameters to fully predict outcomes in PDAC [22], the observed association between *MEAK7* expression and survival warrants further investigation as a potential source of complementary molecular information in prognostic research. Together with the observed upregulation of *MEAK7* in early-stage PDAC samples, these findings support further investigation of its relationship with disease biology and clinical outcomes in independent, well-annotated cohorts.

Analysis of the tumor immune microenvironment revealed that *MEAK7* expression was significantly associated with several immune and stromal cell populations after Benjamini–Hochberg false discovery rate correction. Positive correlations were observed with B cells, CD8^+^ T cells, neutrophils, M0 macrophages, cancer-associated fibroblasts (CAFs), eosinophils, and CD274 (PD-L1), whereas negative correlations were identified with CD4^+^ T cells, monocytes, and natural killer (NK) cells. Although these associations remained statistically significant after correction for multiple testing, the correlation coefficients were generally weak to moderate, indicating modest effect sizes. Therefore, these findings should be interpreted as evidence of statistical association rather than strong biological dependence or direct regulation. Overall, the mixed pattern of positive and negative correlations suggests that elevated *MEAK7* expression is associated with broader alterations in the PDAC tumor microenvironment rather than selective enrichment of a single immune-cell lineage.

Among the associations observed, the positive correlation with cancer-associated fibroblasts (CAFs) is particularly noteworthy. Stromal fibroblasts constitute a major cellular component of pancreatic ductal adenocarcinoma (PDAC) and are integral to extracellular matrix deposition, desmoplasia, immune exclusion, and therapeutic resistance [23]. The modest correlation coefficient identified in this study does not substantiate a direct regulatory function of MEAK7 in fibroblast activation or stromal remodeling. Rather, *MEAK7* expression might merely indicate broader transcriptional programs linked to stromal-rich tumors, a hypothesis necessitating experimental validation. Likewise, the positive correlation between *MEAK7* expression and CD274 (PD-L1) may suggest an association with a more immunoregulatory tumor microenvironment [24]. Given the relatively weak observed correlation and the failure of immune-related survival analyses to demonstrate significant prognostic stratification after multiple-testing correction, these findings do not substantiate the conclusion that MEAK7 defines a clinically meaningful immune-checkpoint phenotype or predicts responsiveness to immune checkpoint inhibition. Future research integrating transcriptomic, proteomic, and functional analyses is necessary to determine whether this association reflects direct biological regulation or indirect co-expression within the tumor microenvironment. Although several statistically significant correlations were observed between *MEAK7* expression and immune or stromal components, survival analyses did not show that immune-cell infiltration significantly altered the prognostic impact of MEAK7. Among patients with high *MEAK7* expression, CD4^+^ T-cell infiltration had the lowest nominal *p* value for overall survival; however, this association lost significance after Benjamini–Hochberg correction (FDR = 0.442). Similarly, no immune or stromal component remained significantly associated with disease-specific survival following adjustment for multiple comparisons. These findings suggest that the observed survival differences should be considered exploratory rather than confirmatory and highlight the necessity of controlling for false-positive findings in high-dimensional immune analyses.

The present analyses utilized predefined immune infiltration estimates and subgroup survival analyses within TIMER 3.0, rather than employing patient-level multivariable Cox regression models. As a result, a formal statistical interaction between *MEAK7* expression and immune-cell infiltration could not be assessed. To demonstrate that immune infiltration modifies the prognostic effect of MEAK7, patient-level Cox proportional hazards models incorporating a MEAK7 × immune-cell infiltration interaction term, along with adjustments for relevant clinicopathological covariates, would be necessary. Therefore, the current findings should be regarded as hypothesis-generating rather than as evidence of a causal interaction between MEAK7 and the immune microenvironment.

Overall, these results indicate that *MEAK7* expression is associated with the complex immune–stromal landscape of PDAC but does not define a distinct immune subtype or independently stratify patient survival based on immune-cell infiltration. Given the observational nature of transcriptomic correlation analyses, further investigations integrating spatial transcriptomics, single-cell sequencing, functional experiments, and clinically annotated patient cohorts are required to ascertain whether MEAK7 actively contributes to immune–stromal remodeling or merely serves as a surrogate marker of broader molecular alterations within the PDAC tumor microenvironment.

STRING identified high-confidence functional associations between MEAK7 and several subunits of the V-ATPase complex, specifically ATP6V1A, ATP6V1C1, ATP6V1D, and ATP6V1H, with combined scores of 0.930, 0.925, 0.926, and 0.925, respectively. This finding suggests that MEAK7 may play a role in biological processes such as cellular pH regulation, vesicular transport, and metabolic adaptation. Previous studies have reported that increased vacuolar-ATPase (v-ATPase) expression and loss of cellular polarity in pancreatic cancer are associated with an invasive tumor phenotype, and that plasma membrane–localized V-ATPase, together with cortactin, may support tumor cell migration and invasion by modulating matrix metalloproteinase activation, particularly MMP-9 [25,26]. In addition, V-ATPase subunits, such as ATP6V1E and ATP6V0C, have been reported to correlate with tumor stage in pancreatic ductal adenocarcinoma [25]. Given the hypoxic and metabolically challenging tumor microenvironment of PDAC, V-ATPase-related processes may be relevant for tumor cell adaptation and survival [27]. In this context, STRING analysis has revealed a functional association network that connects MEAK7 with several proteins related to V-ATPase, such as ATP6V1A, ATP6V1C1, ATP6V1D, ATP6V1H, ATP6V0C, ATP6V0E1, ATP6AP1, ATP6AP2, ATP6V1G1, and RNASEK, all with high combined confidence scores. These results suggest that MEAK7 is functionally linked to proteins involved in V-ATPase-related pathways. However, it is important to note that the STRING database does not confirm direct physical interactions or a central regulatory role for MEAK7. RNASEK also demonstrated one of the highest STRING functional association scores with MEAK7. Previous studies have identified RNASEK as a survival-associated biomarker in pancreatic cancer [28]. Although this observation does not indicate a direct biological interaction, it provides an additional rationale for investigating the potential biological relationship between MEAK7 and RNASEK in future experimental studies. In this study, *MEAK7* expression was positively associated with several PDAC-related genes, including *CTTN*, *CDCP1*, *SMARCA4*, *SFN*, and *PALB2*, suggesting that *MEAK7* may be embedded within a broader molecular network relevant to PDAC biology. Among these genes, CTTN encodes cortactin, a cytoskeletal regulatory protein associated with tumor progression and invasive behavior in pancreatic cancer. Given the previously discussed relationship between V-ATPase activity and cortactin-mediated invasion, the observed association between MEAK7, V-ATPase-related components, and CTTN may indicate a possible link between *MEAK7* expression and invasion-related molecular programs in PDAC. However, this relationship should be interpreted as hypothesis-generating and requires further experimental validation.

Furthermore, high *CDCP1* expression in PDAC is associated with poor survival outcomes and, as a targetable surface receptor, has been shown to influence tumor function and growth in PDAC models [29]. Mutations and disruptions in *SMARCA4*, a component of the SWI/SNF complex, have been documented in pancreatic cancer. Abnormalities in the SWI/SNF complex have been associated with an aggressive phenotype and treatment sensitivity in pancreatic tumors [30]. The positive association between *MEAK7* and *PALB2*, a pancreatic cancer susceptibility gene involved in DNA damage repair, further suggests that MEAK7 may be connected to molecular contexts involving genomic stability [31]. Overall, these correlation-based findings support the possibility that *MEAK7* is associated with multiple PDAC-relevant biological processes, including invasion-related signaling, epigenetic regulation, and DNA damage response pathways, rather than representing an isolated molecular alteration.

It has been reported that miR-135b-5p is elevated in PDAC tissues and is associated with poor prognosis and oncogenic behavior [32], whereas miR-149-3p may exhibit tumor-suppressing properties [33]. Conversely, hsa-miR-582-5p, which was identified as negatively associated with *MEAK7*, and lncRNA *UCA1*, which exhibited a positive correlation with *MEAK7*, play oncogenic roles in pancreatic cancer and may represent novel therapeutic targets for this disease. It has been suggested that UCA1 exerts its oncogenic role by inhibiting hsa-miR-582-5p [34]. These findings raise the possibility that MEAK7 may be associated with a UCA1/miR-582-5p–related regulatory context in PDAC; however, functional studies are required to determine whether MEAK7 participates directly in this post-transcriptional network. While the identified lncRNA–miRNA–MEAK7 associations align with a potential ceRNA regulatory framework, the current findings must be interpreted with caution. Our analyses rely on publicly available transcriptomic datasets and correlation analyses, thus supporting only a putative regulatory relationship rather than a definitive ceRNA mechanism. Establishing a bona fide ceRNA network necessitates direct evidence of lncRNA–miRNA binding, confirmation that the candidate miRNA directly targets the *MEAK7* transcript, appropriate inverse correlation patterns among the interacting molecules, and additional statistical approaches such as partial-correlation, mediation, or conditional-dependence analyses. Moreover, experimental validation through molecular and cellular assays is essential before mechanistic conclusions regarding the proposed ceRNA axis can be drawn. Consequently, the regulatory relationships proposed in this study should be considered hypothesis-generating rather than conclusive mechanistic evidence.

The external GEO analyses provided supportive evidence that increased *MEAK7* expression is detectable in PDAC, including early-stage disease. However, the relationship between *MEAK7* expression and metastatic disease was not consistent across datasets. TNMplot indicated lower *MEAK7* expression in metastatic tissues than in primary tumors, whereas GSE71729 showed the opposite pattern, with significantly higher expression in metastatic samples than in primary tumors. This discrepancy may reflect the biological heterogeneity of metastatic progression, which can be influenced by the patient population, genetic background, metastatic site, lymph node involvement, and tumor microenvironmental context [35]. This discordance is important and precludes inference of a uniform relationship between *MEAK7* expression and metastatic progression. Differences in cohort composition, tissue source, metastatic-site distribution, analytical platform, preprocessing, and normalization may contribute to the observed heterogeneity. Moreover, the TNMplot metastatic group contained only 17 samples, and the available analyses were cross-sectional rather than longitudinal comparisons of matched primary and metastatic lesions. Accordingly, the relationship between MEAK7 and metastatic progression remains unresolved and requires investigation in larger, clinically annotated cohorts containing matched primary and metastatic specimens.

Collectively, the findings of this study suggest that MEAK7 may be linked to a context-dependent molecular network in PDAC, potentially involving tumor progression, epigenetic modulation, and tumor microenvironmental interactions. Rather than being associated with a single dominant mechanism, MEAK7 appears to intersect with multiple biological processes, reflecting the complex and heterogeneous nature of PDAC. Some analyses yielded non-significant or dataset-dependent results, particularly regarding mutation-related associations and immune infiltration. These variations may reflect the biological heterogeneity of PDAC, as well as differences in sample composition, analytical platforms, and normalization strategies. Nonetheless, to substantiate these findings, pancreatic intraepithelial neoplasia-focused studies and further validation analyses of independent clinical cohorts are needed.

### Limitations

This study was based on the retrospective bioinformatic evaluation of transcriptomic, proteomic, survival, and immune data obtained through multiple publicly accessible datasets and analytical platforms. Consequently, the findings are associative, exploratory, and hypothesis-generating rather than causal or clinically validated. Importantly, the use of multiple web-based platforms should not be interpreted as providing independent validation, because several platforms interrogate overlapping or partially overlapping underlying datasets, particularly TCGA-derived cohorts. Accordingly, concordant findings obtained through different interfaces or analytical platforms using overlapping source data were considered complementary or methodologically concordant observations rather than independent replication. Greater evidential weight was therefore placed on analyses performed in separately sourced GEO cohorts; however, these retrospective datasets also differ in cohort composition, tissue source, analytical platform, preprocessing, and normalization and should be regarded as external supportive assessments rather than definitive clinical validation. In addition, the metastatic expression findings were discordant between TNMplot and GSE71729, with opposite directions of *MEAK7* expression change between primary and metastatic tissues. This inconsistency, together with differences in cohort composition and the absence of matched longitudinal primary–metastatic specimens, prevents conclusions regarding the direction of *MEAK7* expression change during metastatic progression.

The proposed lncRNA–miRNA–MEAK7 regulatory network was derived solely from bioinformatic analyses. While the observed expression and correlation patterns indicate a potential ceRNA relationship, they do not confirm a direct regulatory mechanism. This study did not assess direct binding between the candidate lncRNAs and miRNAs, the direct interaction of the candidate miRNAs with the MEAK7 transcript, or causal regulatory relationships through partial-correlation, mediation, or conditional-dependence analyses. Furthermore, the proposed regulatory axis has not been experimentally validated. Consequently, the identified ceRNA network should be regarded as a hypothesis-generating model that offers a framework for future mechanistic and experimental studies, rather than as definitive evidence of a functional regulatory pathway.

A significant limitation of this study is the reliance on transcriptomic, proteomic, survival, and immune infiltration analyses derived from independent publicly available datasets, rather than matched multi-platform molecular profiles from the same patient cohort. As a result, this research constitutes a multi-platform bioinformatic analysis rather than a patient-level multi-omics study, with the various molecular layers serving as complementary sources of evidence. Furthermore, since all analyses are retrospective and based on expression correlations and database-derived associations, they do not establish causal mechanisms underlying the initiation or progression of pancreatic cancer. Consequently, the identified associations should be regarded as exploratory and hypothesis-generating. Although MEAK7 exhibited consistent expression and clinical associations across multiple datasets, these findings alone are insufficient to endorse its use as an early-detection or clinically applicable diagnostic biomarker. Validation of its biological and clinical significance will require prospective patient cohorts and functional experimental studies. Therefore, these findings should be interpreted as complementary evidence rather than patient-level biological validation.

A further limitation is that the survival analyses were performed using the publicly available KM Plotter platform and therefore depended on its predefined analytical workflow, rather than on an independently constructed and validated Cox regression model. While multivariable Cox regression was employed using the variables available on the DoSurvive platform, the dataset lacks several clinically significant prognostic factors, such as treatment modality, surgical resection status, tumor grade, molecular subtype. Therefore, the independent prognostic value of MEAK7 should be interpreted solely within the context of the limited covariates included in the available multivariable model and should not be regarded as evidence of independence from all established clinical prognostic factors. Additionally, the DoSurvive platform does not offer detailed model diagnostics, proportional hazards testing, or information on missing observations, precluding independent evaluation of these aspects in the current study. Future research utilizing well-annotated clinical cohorts with comprehensive clinicopathological and treatment-related variables is necessary to validate the prognostic significance of MEAK7.

Furthermore, although MEAK7’s functional association with V-ATPase components, its relationships with miRNA/lncRNA, and its association with the immune microenvironment were identified in silico, correlation-based analyses could not establish direct immune modulation or mechanistic interactions within the tumor microenvironment. Finally, the absence of experimental validation is a significant limitation of this study, and further in vitro and in vivo studies, including cell line or organoid models, gene silencing/overexpression experiments, protein-level validation, and mechanistic analyses, are necessary to support the biological and translational relevance of the proposed regulatory network. Despite these limitations, our findings provide a hypothesis-generating framework for understanding the potential role of MEAK7 in PDAC biology and support the need for further experimental studies to validate its molecular and functional relevance.

The present study relied largely on publicly available bioinformatic resources. For several web-based analyses, some preprocessing and internal computational steps were platform-defined and were not directly accessible to end users. In contrast, the GSE62452 ROC analyses were conducted using user-defined analytical workflows, and their settings and outputs were documented in the manuscript and supporting files. All accessible analytical parameters, statistical procedures, database versions, and access dates have been comprehensively documented in the manuscript and Supplementary Materials to improve reproducibility.

## 4. Materials and Methods

To improve the reproducibility of this study, Supplementary Table S2 summarizes the analytical workflow, including the input data, user-defined parameters, statistical methods, and outputs for each analytical platform. Most database-based analyses were conducted using publicly available web-based resources, for which certain preprocessing steps and computational pipelines were implemented internally and were not directly accessible to end users. In contrast, the exploratory ROC analyses were performed locally using jamovi. Where available, platform-specific settings, statistical parameters, software and database versions, access dates, and relevant output files were documented throughout the manuscript and Supplementary Materials.

Multiple-testing procedures were defined according to the structure of each analytical domain rather than by applying a single study-wide correction. Where applicable, Benjamini–Hochberg false discovery rate correction or the relevant platform-specific multiple-comparison procedure was used, whereas results based only on nominal *p* values were explicitly identified and interpreted as exploratory. Analyses not involving families of *p*-value-based hypothesis tests were not subjected to additional multiplicity correction.

Additional secondary exploratory analyses, including machine-learning-based tissue classification [36], in silico CRISPR/Cas9 gRNA prioritization [37], and pharmacogenomic drug-sensitivity associations, are provided in the Supplementary Methods and Results, together with the corresponding methodological details, results, figures, tables, and interpretations.

### 4.1. Data Acquisition and Gene Expression Analysis

The expression profile of *MEAK7* in pancreatic adenocarcinoma (PAAD) was examined using RNA sequencing data from The Cancer Genome Atlas (TCGA-PAAD) and Genotype-Tissue Expression (GTEx) projects. Differential expression analysis between tumor and normal pancreatic tissues was conducted using GEPIA3 (https://gepia3.bioinfoliu.com/ accessed on 24 January 2026), a web-based analytical platform. Analyses in GEPIA3 were performed with default parameters, employing |log2 fold change| ≥ 1 and *p* < 0.05 as the criteria for statistical significance [38]. Differential gene expression analysis was performed using the GEPIA3 web server, which implements DESeq2 methodology. Statistical significance was assessed using the Wald test, and *p*-values were adjusted for multiple comparisons using the Benjamini–Hochberg false discovery rate method. The values were presented as log2(TPM + 1).

The Human Protein Atlas (HPA) facilitated a descriptive evaluation of MEAK7 protein expression in both normal pancreatic and pancreatic cancer tissues [39]. Immunohistochemical images and platform annotations, generated using the HPA041020 antibody, were examined concerning staining intensity, cellular localization, and distribution pattern, following the standardized HPA evaluation system. Protein staining was categorized according to the platform’s classifications: not detected, low, medium, and high expression. Based on the HPA antibody-validation data, HPA041020 was designated as “Approved” for immunohistochemistry. The pancreatic cancer series comprised 11 cases, and normal pancreatic tissue images and annotations from the HPA Tissue Atlas were also assessed. The HPA annotations were utilized as reported, without independent digital image quantification, rescoring, or additional statistical analysis. Consequently, the HPA findings were interpreted as complementary descriptive evidence at the protein level.

The expression levels of MEAK7 protein in pancreatic adenocarcinoma were examined using proteomic data sourced from the Clinical Proteomic Tumor Analysis Consortium (CPTAC) database via UALCAN. The analyses employed normalized protein expression data derived from liquid chromatography–tandem mass spectrometry (LC–MS/MS)-based methodologies. Proteomic subgroup analyses were conducted utilizing the UALCAN CPTAC portal. This platform processes CPTAC mass spectrometry data through within-sample log_2_ normalization, followed by Z-score transformation, where protein expression values indicate the number of standard deviations from the median across samples. All statistical comparisons and corresponding *p*-values presented in this study were automatically generated by the UALCAN analytical pipeline and directly verified against the platform output. UALCAN performs pairwise comparisons using a two-sample *t*-test and reports Z-normalized expression values and *p* values. The platform does not provide integrated multi-group ANOVA, post hoc correction, effect-size estimates, or confidence intervals.

The proteomic expression of MEAK7 was independently analyzed according to patient sex and mTOR pathway status. Normal pancreatic tissue samples were used as reference samples. Gender-based analyses were conducted by comparing male and female patients. In the mTOR pathway analysis, the samples were classified as mTOR pathway–altered and unaltered or other (wild type). All analyses were conducted using the CPTAC data portal, and the results were visualized using box plots and individual sample points to illustrate the distribution of MEAK7 protein expression across each subgroup. The number of samples (*n*) in each group is indicated in each panel [40].

### 4.2. Exploratory ROC-Based Tissue Expression Discrimination Analysis

An exploratory ROC analysis was performed to characterize the ability of *MEAK7* expression to distinguish primary PDAC tumor tissue from adjacent non-tumor pancreatic tissue using the GSE62452 dataset. ROC analyses were performed using Jamovi version 2.3.28. The GSE62452 dataset is publicly available through the NCBI Gene Expression Omnibus database (https://www.ncbi.nlm.nih.gov/geo/query/acc.cgi?acc=GSE62452, accessed on 1 August 2026). This dataset comprises 69 primary PDAC tumor samples and 61 adjacent non-tumor pancreatic tissue samples profiled using the Affymetrix Human Gene 1.0 ST Array platform (GPL6244) [41]. The normalized expression matrix deposited in the Gene Expression Omnibus (GEO) database was used. According to the GEO record, the microarray data were generated in two experimental sets, and batch effects between these sets were removed by the original investigators using Partek Genomic Suite before the merged normalized matrix was deposited.

Expression values for *MEAK7* and the prespecified PDAC driver genes *KRAS*, *TP53*, *CDKN2A*, and *SMAD4* were included in the analysis. The driver genes were used as biologically relevant contextual comparators and not as clinically validated diagnostic reference standards.

For the ROC analyses, PDAC tumor samples were defined as the positive class and adjacent non-tumor pancreatic samples as the negative class. For each gene, the area under the ROC curve (AUC), two-sided 95% confidence interval (CI), optimal expression cut-off, sensitivity, and specificity were calculated. Standard errors of the AUCs were estimated using the nonparametric DeLong method, and the 95% CIs were calculated using the normal approximation as follows: AUC ± 1.96 × SE_DeLong. Gene-specific optimal cut-offs were defined as the values maximizing the Youden index, and sensitivity and specificity were reported at the corresponding cut-offs.

Because cut-off selection and performance estimation were conducted within the same cohort without independent hold-out or external validation of the derived thresholds, the analysis was interpreted as an exploratory assessment of tissue expression discrimination rather than an evaluation of clinical diagnostic performance.

### 4.3. Gene Expression Analysis (Tumor–Normal–Metastatic)

The expression levels of *MEAK7* in normal, primary tumor, and metastatic tissues in pancreatic cancer were assessed using TNMplot (https://tnmplot.com/analysis/, accessed on 24 January 2026), an online analytical platform that evaluates publicly available transcriptomic data. This platform facilitates comparative gene expression analysis between normal, tumor, and metastatic samples by combining RNA sequencing and DNA microarray data analysis.

In this analysis, normal pancreatic tissue, primary tumor tissue, and available metastatic samples associated with pancreatic cancer were examined. The expression levels of *MEAK7* were compared among the three groups. The statistical significance of the differences between groups was assessed using the Kruskal–Wallis test. Pairwise comparisons were performed using Dunn’s post hoc multiple-comparison test as implemented by the platform. The results were reported as DESeq2-normalized and scaled expression values, with a *p*-value < 0.05 considered statistically significant. To elucidate the potential molecular interactions of MEAK7 in pancreatic cancer tumor tissue, we analyzed the genes correlated with *MEAK7* expression in tumor samples using the TNM Plot database. This analysis assessed the relationship between *MEAK7* expression levels and the expression of other genes using Spearman’s rank correlation. Genes exhibiting positive or negative correlations that exceeded a specified threshold were considered to be statistically significant. These genes were subsequently assessed as candidate genes potentially involved in the biological processes or signaling pathways shared with *MEAK7*. Correlation analyses were conducted exclusively on tumor tissue samples, and the results are presented in tables and graphs.

### 4.4. Survival Analysis

The Kaplan–Meier plotter (https://kmplot.com/analysis/, accessed on 24 January 2026) was used to assess the correlation between the expression of all genes (mRNA, miRNA, protein, and DNA) and survival across more than 40,000 samples from 21 tumor types [42]. Survival analysis was performed to evaluate the prognostic effects of *MEAK7* expression levels. Patients were divided into high- and low-expression groups according to the auto-selected best cutoff. This option evaluates eligible cutoff values between the lower and upper quartiles of gene expression and selects the optimal cutoff based on Cox proportional hazards regression. To address the issue of multiple hypothesis testing introduced by cutoff optimization, the platform automatically implements the Benjamini–Hochberg false discovery rate (FDR) correction. Kaplan–Meier survival curves were constructed, and intergroup differences were evaluated using the log-rank test. Hazard ratios (HRs) with 95% confidence intervals (CIs) were determined using a univariate Cox proportional hazards model. JetSet best probes were used for microarray-based analyses. In these analyses, gene expression data from pertinent cancer cohorts were integrated with clinical survival information to produce Kaplan–Meier survival curves, and differences between groups were evaluated using the log-rank test. Statistical significance was set at *p* < 0.05.

### 4.5. DoSurvive-Based Survival and Cox Regression Analysis

To assess the association between gene expression and survival in pancreatic adenocarcinoma (PAAD), the Disease Outcome & Survival Visualization (DoSurvive) web-based database was employed (https://dosurvive.lab.nycu.edu.tw/gene# accessed on 24 January 2026) [43]. This platform functions as a tool for visualizing the relationships between gene expression and clinical outcomes across The Cancer Genome Atlas (TCGA), Gene Expression Omnibus (GEO), and other publicly accessible genomic datasets. Survival analyses were performed using the DoSurvive web-based platform. Patients were stratified into high- and low-expression cohorts based on median *MEAK7* expression. Kaplan–Meier survival curves were generated, and differences between the groups were evaluated using the log-rank test. Multivariate Cox proportional hazards regression analyses were performed to determine the independent prognostic significance of *MEAK7* expression after adjusting for age, sex, and tumor stage.

### 4.6. TIMER 3.0-Based Immune Infiltration Analysis

Tumor immune infiltration was evaluated using the TIMER 3.0 web platform (http://timer.cistrome.org; accessed on 24 January 2026), which enables immune deconvolution using multiple algorithms that are optimized for TCGA-PAAD datasets. Gene expression matrices were analyzed using integrated immune estimation modules, including TIMER, EPIC, MCP-counter, CIBERSORT, CIBERSORT-ABS, QUANTISEQ, xCell, and TIDE. For each gene of interest, the correlation between its expression and the abundance of immune cells (B cells, CD4^+^ T cells, CD8^+^ T cells, macrophages, neutrophils, and dendritic cells) was calculated using the partial Spearman correlation, with adjustments for tumor purity. Statistical significance was determined using the default parameters provided by TIMER 3.0. Immune infiltration estimates and corresponding correlation coefficients were exported directly from the platform for subsequent visualization and interpretation [44]. The joint effects of MEAK7 expression and immune/stromal infiltration on overall survival (OS) and disease-specific survival (DSS) were evaluated in the TCGA-PAAD cohort using TIMER3. Patients were divided into four groups according to MEAK7 expression and infiltration levels, and Group 4 was compared with Group 3. Hazard ratios and nominal *p* values were obtained from the platform.

To control the false-positive rate arising from multiple comparisons, Benjamini–Hochberg false discovery rate correction was applied separately within each prespecified family of related analyses. Immune-infiltration correlations, overall-survival immune subgroup comparisons, and disease-specific-survival immune subgroup comparisons were treated as distinct testing families. Results with an FDR-adjusted *p* value below 0.05 were considered statistically significant.

### 4.7. STRING Database-Based Functional Association Analysis of MEAK7

STRING-derived functional association networks for MEAK7 were generated using the STRING database (version 12.0; https://string-db.org; accessed 24 January 2026). Interactors were retrieved for Homo sapiens, and only high-confidence interactions (minimum interaction score of 0.700) were included. First-shell interactors were selected, and edges supported by experimental evidence or curated databases were prioritized for analysis. The resulting functional association network was visualized and exported using the publication-ready output settings provided by the STRING platform [45,46].

### 4.8. Prediction of MEAK7 miRNA Interactions Using TargetScan 8.0 and miRDB

Predicted miRNA–mRNA interactions were explored using the TargetScanHuman database (Release 8.0; https://www.targetscan.org; accessed 24 January 2026). This version of TargetScan identifies potential binding sites in mammalian transcripts by detecting canonical 8mer, 7mer, and 6mer seed matches and prioritizing candidate miRNAs using an updated context++ scoring approach. Release 8.0 incorporates an enhanced biochemical model that refines target ranking by integrating seed pairing, site accessibility, 3′-untranslated region (UTR) features and evolutionary conservation. Family-level prediction files were downloaded, and for each gene of interest, the cumulative weighted context++ score (CWCS) and, when available, the probability of conserved targeting (PCT) were extracted. As lower (more negative) CWCS values indicate greater predicted inhibitory activity, these metrics were used as the basis for interpreting the miRNA regulatory strength [47].

To complement the TargetScan analysis, miRNA target predictions were retrieved from miRDB (http://mirdb.org; accessed on 24 January 2026), a machine learning-based resource trained on high-throughput CLIP-Seq experimental datasets. miRDB assigns each predicted interaction a numerical “target score” (ranging from 50 to 100), which reflects the confidence in miRNA binding based on its MirTarget computational model. For each candidate gene, miRNAs with a target score of ≥80 were considered high-confidence regulators. All predictions were downloaded using the default parameters, and overlapping miRNAs identified by both TargetScan and miRDB were prioritized for downstream analyses [48].

### 4.9. LncRNAs Associated with PAAD Identified Through the LncRNADisease Database

The lncRNADisease database (http://www.cuilab.cn/lncrnadisease, accessed on 24 January 2026) was used to identify long non-coding RNAs associated with PAAD. Disease-associated long non-coding RNAs (lncRNAs) were identified through the LncRNADisease database, a manually curated repository that integrates experimentally validated associations between lncRNAs and human diseases. This database compiles lncRNA–disease relationships by systematically curating literature, incorporating evidence from differential expression analyses, functional experiments, molecular interaction studies, and clinically relevant observations. Each association is substantiated by corresponding published evidence and assigned a confidence level based on the robustness of experimental validation. To identify lncRNAs associated with pancreatic cancer, the LncRNADisease database was queried using “pancreatic cancer” as the disease term (Supplementary Table S3). Candidate lncRNAs previously reported in association with pancreatic cancer were retrieved and subsequently screened for potential interactions with the identified MEAK7.

This resource compiles experimentally validated associations between lncRNAs and human diseases and provides computational tools for predicting potential lncRNA–disease relationships. Furthermore, the platform integrates multilayer interaction data for each lncRNA, including its interactions with DNA, RNA, proteins and miRNAs [49].

### 4.10. ENCORI-Based Expression Correlation Analysis of MEAK7 with Candidate miRNAs and lncRNAs

Expression correlations between *MEAK7 (TLDC1)* and selected candidate miRNAs and lncRNAs were evaluated using the ENCORI database, also known as starBase (https://starbase.sysu.edu.cn; accessed 24 January 2026). Candidate miRNAs were selected from the overlapping predictions obtained using TargetScanHuman 8.0 and miRDB, whereas candidate lncRNAs were selected from pancreatic cancer-associated lncRNAs identified through the LncRNADisease database. Correlation analyses were performed in the TCGA-PAAD cohort comprising 178 samples. According to the platform-generated outputs, *TLDC1* and lncRNA expression levels were represented as log2(FPKM + 0.01), whereas miRNA expression levels were represented as log2(RPM + 0.01). Correlation coefficients (r), regression lines, and corresponding two-sided *p* values were obtained directly from the ENCORI platform. A *p* value < 0.05 was considered statistically significant. These analyses assessed expression co-variation and were not interpreted as evidence of direct molecular binding or causal regulation [50].

### 4.11. External GEO Cohorts and Differential Expression Analysis of MEAK7

Gene expression datasets were obtained from the Gene Expression Omnibus (GEO) repository (accessed 24 January 2026) [51]. Three externally sourced GEO cohorts, GSE62165, GSE183795, and GSE71729, were used to assess *MEAK7* expression across different pancreatic tissue contexts. GSE62165 was generated using the GPL13667 Affymetrix Human Genome U219 Array [52], GSE183795 using the GPL6244 Affymetrix Human Gene 1.0 ST Array [53] and GSE71729 using the GPL20769 Agilent Whole Human Genome Microarray 4×44K G4112F platform [54]. Processed expression data available through GEO2R were analyzed using the limma framework implemented in R; no additional local preprocessing of raw microarray files was performed. For differential-expression analyses, nominal *p* values were adjusted for multiple comparisons using the Benjamini–Hochberg false discovery rate (FDR) procedure. Both nominal and FDR-adjusted *p* values were reported where available, with FDR-adjusted *p* < 0.05 considered statistically significant.

For GSE62165, 38 early-stage PDAC tissue samples and 13 non-tumor pancreatic control tissue samples were included. The comparison was performed at the group level rather than as a matched tumor–control analysis. The contrast was defined as early-stage PDAC versus control, such that positive log2 fold-change values indicated higher expression in the PDAC group. The samples were designated as early-stage PDAC according to the clinical annotation provided with the original GEO dataset; no additional retrospective stage reclassification was performed in the present study. When multiple probes mapped to the same gene symbol, the probe with the lowest FDR-adjusted *p* value was selected for gene-level reporting. Five probes mapped to KIAA1609, and probe 11730349_a_at, which showed the lowest Benjamini–Hochberg-adjusted *p* value, was selected as the representative probe. Complete probe-level results are provided in Supplementary Table S4.

For GSE183795, *MEAK7* expression was assessed in 139 PDAC tumor tissues and 102 adjacent non-tumor pancreatic tissues. Differential-expression analysis was performed using the processed GEO expression data through GEO2R/limma, with Benjamini–Hochberg FDR correction for multiple testing. The analysis was interpreted as a group-level tumor-versus-adjacent non-tumor comparison. Pairing information was not incorporated into the present statistical analysis; therefore, the findings were not interpreted as a paired within-patient comparison.

For GSE71729, *MEAK7* expression was evaluated across 46 normal pancreatic tissue samples, 145 primary PDAC tissue samples, and 61 metastatic PDAC tissue samples using the processed expression data available through GEO2R. Three predefined contrasts were examined: primary tumor versus normal tissue, primary tumor versus metastatic tissue, and metastatic tissue versus normal tissue. Differential-expression statistics were obtained using the limma framework with Benjamini–Hochberg FDR correction. The primary and metastatic groups were analyzed as cross-sectional tissue groups rather than as longitudinally matched primary–metastatic samples from the same patients. Detailed metastatic-site stratification was not incorporated into the present analysis because this information was not consistently available for the analyzed comparison.

### 4.12. External Transcriptomic Assessment of MEAK7 Expression Using EMBL-EBI Expression Atlas

External transcriptomic analysis of *MEAK7* expression was performed using the EMBL-EBI Expression Atlas database (https://www.ebi.ac.uk/gxa; accessed on 17 March 2026). Baseline *MEAK7* expression was examined exclusively in pancreatic adenocarcinoma cell lines available within the database [55]. The gene symbol “MEAK7” was queried under the condition “pancreatic adenocarcinoma” (Homo sapiens), and the expression patterns were evaluated based on platform-defined expression levels.

## 5. Conclusions

This exploratory multi-platform bioinformatic study identified increased *MEAK7* expression in PDAC and demonstrated associations with survival outcomes, immune and stromal features, co-expression patterns, and broader molecular functional association networks. Analyses performed across different web-based platforms were interpreted as complementary, where the underlying source datasets may overlap and were not considered independent validation. Assessments in separately sourced GEO cohorts provided additional supportive evidence for altered *MEAK7* expression, although metastatic expression patterns varied across datasets. Externally sourced GEO cohorts provided additional supportive evidence for increased *MEAK7* expression in PDAC, including early-stage tumors. However, the opposite metastatic expression patterns observed in TNMplot and GSE71729 indicate that the relationship between *MEAK7* expression and metastatic progression remains unresolved.

Network-based, regulatory RNA and immune infiltration analyses further generated testable hypotheses regarding the potential biological context of MEAK7 in PDAC. These computational associations do not establish causal mechanisms or diagnostic, prognostic, or therapeutic utility. Overall, the present findings provide a hypothesis-generating framework for future investigation of MEAK7 in PDAC. Experimental validation and prospectively designed studies in independent, clinically well-characterized patient cohorts are required to determine its biological significance and any potential future clinical relevance.

MEAK7 shows potential prognostic relevance in PDAC. However, the varied metastatic expression patterns observed across independent datasets suggest that its role in disease progression warrants further investigation through well-characterized patient cohorts and functional studies. These findings provide a hypothesis-generating framework for understanding the potential involvement of MEAK7 in PDAC-associated molecular processes, including transcriptomic, epigenetic, post-transcriptional, and tumor microenvironmental contexts. Further validation in independent patient cohorts and functional experimental studies is required to clarify the biological and clinical significance of MEAK7 in PDAC.

## Figures and Tables

**Table 1:** Exploratory tissue expression discrimination of *MEAK7* and selected PDAC driver genes in the GSE62452 cohort.

Gene	AUC (95% Confidence Interval)	Cutoff	Sensitivity (%)	Specificity (%)
*MEAK7*	0.795 (0.719–0.871)	4.279	63.8	85.3
*KRAS*	0.751 (0.667–0.835)	6.038	84.1	60.7
*TP53*	0.694 (0.604–0.784)	5.580	52.2	82.0
*CDKN2A*	0.579 (0.479–0.679)	4.283	59.4	60.7
*SMAD4*	0.742 (0.658–0.826)	7.182	75.4	67.2

AUC, area under the receiver operating characteristic curve; CI, confidence interval. Gene-specific cut-offs were determined using the Youden index. The 95% CIs for the AUCs were calculated using the DeLong method.

**Table 2:** Top genes positively correlated with *MEAK7* expression in the TCGA-PAAD cohort.

Rank	Gene	Spearman’s *ρ*	Nominal *p* Value	Benjamini–Hochberg FDR	Functional Annotation
1	CTTN	0.74	3.89 × 10^−41^	7.78 × 10^−40^	Cytoskeleton remodeling/cell migration
2	STAU1	0.73	5.25 × 10^−39^	5.25 × 10^−38^	RNA transport and stability
3	SFN	0.72	7.39 × 10^−38^	4.93 × 10^−37^	Cell-cycle regulation
4	SMARCA4	0.72	3.60 × 10^−37^	1.80 × 10^−36^	Chromatin remodeling
5	SFXN1	0.71	5.51 × 10^−36^	2.20 × 10^−35^	Mitochondrial serine transport
6	GSS	0.71	6.17 × 10^−36^	2.06 × 10^−35^	Glutathione biosynthesis
7	EFNA5	0.71	6.17 × 10^−36^	1.76 × 10^−35^	Cell signaling
8	OPA1	0.71	1.00 × 10^−35^	2.50 × 10^−35^	Mitochondrial dynamics
9	CDCP1	0.71	1.19 × 10^−35^	2.64 × 10^−35^	Cell adhesion/invasion
10	CARS	0.70	1.61 × 10^−35^	3.22 × 10^−35^	Protein translation
11	PALB2	0.70	3.02 × 10^−35^	5.49 × 10^−35^	DNA repair
12	ARFGEF1	0.70	3.91 × 10^−35^	6.52 × 10^−35^	Vesicle trafficking
13	KBTBD2	0.70	4.96 × 10^−35^	7.63 × 10^−35^	Ubiquitination
14	SRD5A1	0.70	6.11 × 10^−35^	8.73 × 10^−35^	Steroid metabolism
15	KDM2A	0.70	1.57 × 10^−34^	2.09 × 10^−34^	Epigenetic regulation
16	BRD8	0.69	9.59 × 10^−34^	1.20 × 10^−33^	Chromatin regulation
17	TUFT1	0.69	1.33 × 10^−33^	1.56 × 10^−33^	Cell survival
18	MTIF2	0.69	2.53 × 10^−33^	2.81 × 10^−33^	Mitochondrial translation
19	STK24	0.69	2.53 × 10^−33^	2.66 × 10^−33^	Protein kinase signaling
20	KCMF1	0.69	3.70 × 10^−33^	3.70 × 10^−33^	Protein ubiquitination

**Table 3:** Significant correlations between *MEAK7* expression and immune cell infiltration and stromal/immune-related components in pancreatic adenocarcinoma (PAAD) after Benjamini–Hochberg FDR correction.

Immune Cell	*ρ*	Nominal *p*	FDR-Adjusted *p*
Monocytes (QUANTISEQ)	−0.294	1.02 × 10^−4^	0.00102
Cancer-associated fibroblasts (EPIC)	0.279	2.25 × 10^−4^	0.00113
CD274 (PD-L1, TIDE)	0.262	5.45 × 10^−4^	0.00142
B cells (TIMER)	0.262	5.69 × 10^−4^	0.00142
M0 macrophages (CIBERSORT)	0.234	2.16 × 10^−3^	0.00432
CD8^+^ T cells (TIMER)	0.217	4.51 × 10^−3^	0.00667
Neutrophils (TIMER)	0.216	4.67 × 10^−3^	0.00667
CD4^+^ T cells (TIMER)	−0.190	1.33 × 10^−2^	0.01663
Eosinophils (CIBERSORT)	0.158	3.91 × 10^−2^	0.04200
NK cells (QUANTISEQ)	−0.156	4.20 × 10^−2^	0.04200

**Table 4:** Summary of overall survival (OS) and disease-specific survival (DSS) analyses according to immune/stromal cell infiltration among patients with high *MEAK7* expression.

Immune/Stromal Component	OS (4 vs. 3) *p*	OS FDR-Adjusted *p*	DSS (4 vs. 3) *p*	DSS FDR-Adjusted *p*
Cancer-associated fibroblasts (EPIC)	0.295	>0.05	0.418	>0.05
CD4^+^ T cells (TIMER)	0.0442	0.4420	0.0519	0.3095
CD8^+^ T cells (TIMER)	0.285	>0.05	0.357	>0.05
CD274 (PD-L1, TIDE)	0.389	>0.05	0.311	>0.05
Eosinophils	0.350	>0.05	0.0619	0.3095
M1 macrophages (CIBERSORT)	0.705	>0.05	0.645	>0.05
Monocytes (quanTIseq)	0.357	>0.05	0.383	>0.05
NK cells (quanTIseq)	0.181	>0.05	0.219	>0.05
Neutrophils (TIMER/CIBERSORT)	0.289	>0.05	0.357	>0.05
B cells (TIMER)	0.430	>0.05	0.501	>0.05

Group 4 represents patients with high *MEAK7* expression and high immune/stromal cell infiltration, whereas Group 3 represents patients with high *MEAK7* expression and low immune/stromal cell infiltration. Nominal *p* values were corrected for multiple comparisons using the Benjamini–Hochberg false discovery rate (FDR) procedure. OS, overall survival; DSS, disease-specific survival; FDR, false discovery rate.

**Table 5:** STRING evidence channels and combined functional-association scores.

Gene-1	Gene-2	Protein Annotation	Interaction	Combined Score
*MEAK7*	*RNASEK*	Ribonuclease kappa	CE ED LC	0.956
*MEAK7*	*ATP6V0E1*	V-type proton ATPase subunit e 1	CE ED	0.930
*MEAK7*	*ATP6V1A*	V-type proton ATPase catalytic subunit A	TM ED CE	0.930
*MEAK7*	*ATP6V0C*	V-type proton ATPase 16 kDa proteolipid subunit	TM ED CE	0.926
*MEAK7*	*ATP6V1D*	V-type proton ATPase subunit D	CE ED	0.926
*MEAK7*	*ATP6AP1*	Renin receptor C-terminal fragment	CE ED	0.925
*MEAK7*	*ATP6V1C1*	V-type proton ATPase subunit C 1	CE ED	0.925
*MEAK7*	*ATP6V1G1*	V-type proton ATPase subunit G 1	CE ED	0.925
*MEAK7*	*ATP6V1H*	V-type proton ATPase subunit H	CE ED	0.925
*MEAK7*	*ATP6AP2*	V-type proton ATPase subunit S1	ED TM	0.925

Abbreviations: TM: Text mining, CE: Co-expression, ED: Experimentally determined, LC: Literature curation.

**Table 6:** miRNAs associated with MEAK7 were identified using a combination of the TargetScanHuman8.0 and miRDB databases.

Predicted miRNAs for MEAK7	
miRDB databases TargetScanHuman8.0	hsa-miR-582-5p, hsa-miR-135b-5p, hsa-miR-135a-5p, hsa-miR-202-3p, hsa-miR-432-5p, hsa-miR-6855-3p, hsa-miR-4513, hsa-miR-3670, hsa-miR-149-3p

**Table 7:** *MEAK7* expression in pancreatic adenocarcinoma cell lines from the Expression Atlas.

Cell Line	CCLE	675 Genentech (PAAD)	Cancer Genome Project	675 Genentech (Tubular)	Overall Expression
AsPC-1	NA	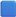 Medium	NA	NA	Medium
BxPC-3	NA	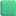 High	NA	NA	High
Capan-2	NA	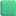 High	NA	NA	High
DAN-G	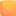 Low	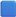 Medium	NA	NA	Medium
HPAC	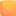 Low	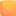 Low	NA	NA	Low
Hs766T	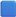 Medium	NA	NA	NA	Medium
HUP-T3	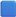 Medium	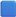 Medium	NA	NA	Medium
HUP-T4	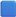 Medium	NA	NA	NA	Medium
KCI-MOH1	NA	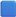 Medium	NA	NA	Medium
MZ1-PC	NA	NA	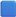 Medium	NA	Medium
PA-TU-8902	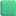 High	NA	NA	NA	High
PA-TU-8988S	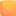 Low	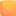 Low	NA	NA	Low
PA-TU-8988T	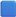 Medium	NA	NA	NA	Medium
Panc 02.03	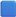 Medium	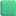 High	NA	NA	High
Panc 02.13	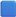 Medium	NA	NA	NA	Medium
Panc 03.27	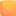 Low	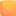 Low	NA	NA	Low
Panc 04.03	NA	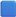 Medium	NA	NA	Medium
Panc 05.04	NA	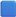 Medium	NA	NA	Medium
Panc 08.13	NA	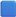 Medium	NA	NA	Medium
PANC-1	NA	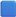 Medium	NA	NA	Medium
PL18	NA	NA	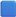 Medium	NA	Medium
PL4	NA	NA	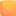 Low	NA	Low
PSN1	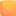 Low	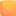 Low	NA	NA	Low
SNU-213	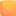 Low	NA	NA	NA	Low
SNU-324	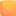 Low	NA	NA	NA	Low
SNU-410	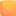 Low	NA	NA	NA	Low
SU.86.86	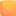 Low	NA	NA	NA	Low
SUIT-2	NA	NA	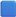 Medium	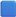 Medium	Medium
SW1990	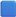 Medium	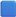 Medium	NA	NA	Medium
TCC-PAN2	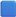 Medium	NA	NA	NA	Medium

Legend: High (green) (≥50 TPM); Medium (blue) (20–49 TPM); Low (yellow) (1–19 TPM); NA (white) (No available data).

## Data Availability

Publicly available data analyzed in this study were obtained from TCGA, GTEx, CPTAC, and GEO datasets GSE62452, GSE62165, GSE71729, and GSE183795. Dataset links, accession numbers, and access dates are provided in the Section 4. The corresponding analytical workflows are summarized in Supplementary Table S2.

## Supplementary Materials

The following supporting information can be downloaded at: https://www.mdpi.com/article/10.3390/ijms27177610/s1.

## Abbreviations

The following abbreviations are used in this manuscript:

Akt	Protein Kinase B
CPTAC	Clinical Proteomic Tumor Analysis Consortium
DoSurvive	Disease Outcome & Survival Visualization
DSS	Disease-Specific Survival
FDR	False Discovery Rate
GEO	Gene Expression Omnibus
GIT1	G protein-coupled receptor kinase-interacting ArfGAP 1
IHC	Immunohistochemical
LC–MS/MS	Liquid Chromatography–Tandem Mass Spectrometry
lncRNA	Long Non-Coding RNA
MEAK7	Mammalian EAK-7
mTOR	Mammalian Target of Rapamycin
OS	Overall Survival
PAAD	Pancreatic Adenocarcinoma
PDAC	Pancreatic Ductal Adenocarcinoma
PFS	Progression-Free Survival
PI3K	Phosphatidylinositol-3 Kinase
TCGA	The Cancer Genome Atlas

## References

[1] Siegel R.L., Kratzer T.B., Wagle N.S., Sung H., Jemal A. (2026). Cancer statistics, 2026. CA Cancer J. Clin..

[2] Stoop T.F., Javed A.A., Oba A., Koerkamp B.G., Seufferlein T., Wilmink J.W., Besselink M.G. (2025). Pancreatic cancer. Lancet.

[3] Stanciu S., Ionita-Radu F., Stefani C., Miricescu D., Stanescu-Spinu I.-I., Greabu M., Totan A.R., Jinga M. (2022). Targeting PI3K/AKT/mTOR Signaling Pathway in Pancreatic Cancer: From Molecular to Clinical Aspects. Int. J. Mol. Sci..

[4] Jamal M.H., Khan M.N. (2025). Developments in pancreatic cancer emerging therapies, diagnostic methods, and epidemiology. Pathol. Res. Pract..

[5] Elsayed M., Abdelrahim M. (2021). The Latest Advancement in Pancreatic Ductal Adenocarcinoma Therapy: A Review Article for the Latest Guidelines and Novel Therapies. Biomedicines.

[6] Iriana S., Ahmed S., Gong J., Annamalai A.A., Tuli R., Hendifar A.E. (2016). Targeting mTOR in pancreatic ductal adenocarcinoma. Front. Oncol..

[7] Ouissam A.J., Hind C., Sami Aziz B., Said A. (2024). Inhibition of the PI3K/AKT/mTOR pathway in pancreatic cancer: Is it a worthwhile endeavor?. Ther. Adv. Med. Oncol..

[8] Driscoll D.R., A Karim S., Sano M., Gay D.M., Jacob W., Yu J., Mizukami Y., Gopinathan A., Jodrell D.I., Evans T.J. (2016). mTORC2 Signaling Drives the Development and Progression of Pancreatic Cancer. Cancer Res..

[9] Nguyen J.T., Ray C., Fox A.L., Mendonça D.B., Kim J.K., Krebsbach P.H. (2018). Mammalian EAK-7 activates alternative mTOR signaling to regulate cell proliferation and migration. Sci. Adv..

[10] Nguyen J.T., Haidar F.S., Fox A.L., Ray C., Mendonça D.B., Kim J.K., Krebsbach P.H. (2019). mEAK-7 Forms an Alternative mTOR Complex with DNA-PKcs in Human Cancer. iScience.

[11] Ohira M., Kitamura M., Iwasaki H., Ohta-Okano H., Tsujii H., Nakamura R., Nakazawa T., Nishiguchi A., Yamamoto M., Osada K. (2025). Collagen Signaling via DDR1 Exacerbates Barriers to Macromolecular Drug Delivery in a 3D Model of Pancreatic Cancer Fibrosis. Small.

[12] Wolde T., Bhardwaj V., Pandey V. (2025). Current Bioinformatics Tools in Precision Oncology. MedComm.

[13] Zhang S., Liu K., Liu Y., Hu X., Gu X. (2025). The role and application of bioinformatics techniques and tools in drug discovery. Front. Pharmacol..

[14] Yay F., Uyaner Kan M. (2026). NECTIN4 (PVRL4) expression in pancreatic cancer patients might be associated with a more unfavorable prognosis, independent of age at diagnosis, sex, and stage: A public microarray and RNA sequencing data based bioinformatics analysis. Comput. Biol. Chem..

[15] Liu X., Chen B., Chen J., Sun S. (2021). A novel tp53-associated nomogram to predict the overall survival in patients with pancreatic cancer. BMC Cancer.

[16] Posta M., Győrffy B. (2023). Analysis of a large cohort of pancreatic cancer transcriptomic profiles to reveal the strongest prognostic factors. Clin. Transl. Sci..

[17] Yuan P., Tang C., Chen B., Lei P., Song J., Xin G., Wang Z., Hui Y., Yao W., Wang G. (2021). miR-32-5p suppresses the proliferation and migration of pancreatic adenocarcinoma cells by targeting TLDC1. Mol. Med. Rep..

[18] Stefanoudakis D., Frountzas M., Schizas D., Michalopoulos N.V., Drakaki A., Toutouzas K.G. (2024). Significance of TP53, CDKN2A, SMAD4 and KRAS in Pancreatic Cancer. Curr. Issues Mol. Biol..

[19] Voutsadakis I.A. (2021). Mutations of p53 associated with pancreatic cancer and therapeutic implications. Ann. Hepato-Biliary-Pancreat. Surg..

[20] Huang L., Guo Z., Wang F., Fu L. (2021). KRAS mutation: From undruggable to druggable in cancer. Signal Transduct. Target. Ther..

[21] Uniyal P., Kashyap V.K., Behl T., Parashar D., Rawat R. (2025). KRAS Mutations in Cancer: Understanding Signaling Pathways to Immune Regulation and the Potential of Immunotherapy. Cancers.

[22] Mori H., Miyake T., Maehira H., Shiohara M., Iida H., Nitta N., Tani M. (2024). Contribution of Immunoscore to Survival Prediction in Pancreatic Ductal Adenocarcinoma. Anticancer. Res..

[23] Zhang T., Ren Y., Yang P., Wang J., Zhou H. (2022). Cancer-associated fibroblasts in pancreatic ductal adenocarcinoma. Cell Death Dis..

[24] Mucileanu A., Chira R., Mircea P.A. (2021). PD-1/PD-L1 expression in pancreatic cancer and its implication in novel therapies. Med. Pharm. Rep..

[25] Chung C., Mader C.C., Schmitz J.C., Atladottir J., Fitchev P., Cornwell M.L., Koleske A.J., Crawford S.E., Gorelick F. (2011). The vacuolar-ATPase modulates matrix metalloproteinase isoforms in human pancreatic cancer. Lab. Investig..

[26] Flinck M., Hagelund S., Gorbatenko A., Severin M., Pedraz-Cuesta E., Novak I., Stock C., Pedersen S.F. (2020). The Vacuolar H^+^ ATPase α3 Subunit Negatively Regulates Migration and Invasion of Human Pancreatic Ductal Adenocarcinoma Cells. Cells.

[27] Chen Z., Han F., Du Y., Shi H., Zhou W. (2023). Hypoxic microenvironment in cancer: Molecular mechanisms and therapeutic interventions. Signal Transduct. Target. Ther..

[28] Hassan Elsheikh N.A., Abaker N.A.A., Almrshoud M.F., Abdalfadil F.A.A., Alfaki M. (2025). Pan-Cancer Analysis Reveals Ribonuclease K (RNASEK) as a Potential Prognostic Biomarker in Pancreatic Cancer and a Diagnostic Indicator Across Multiple Human Cancers. Cureus.

[29] Kryza T., Khan T., Puttick S., Li C., Sokolowski K.A., Tse B.W., Cuda T., Lyons N., Gough M., Yin J. (2020). Effective targeting of intact and proteolysed CDCP1 for imaging and treatment of pancreatic ductal adenocarcinoma. Theranostics.

[30] Shain A.H., Giacomini C.P., Matsukuma K., Karikari C.A., Bashyam M.D., Hidalgo M., Maitra A., Pollack J.R. (2012). Convergent structural alterations define SWItch/Sucrose NonFermentable (SWI/SNF) chromatin remodeler as a central tumor suppressive complex in pancreatic cancer. Proc. Natl. Acad. Sci. USA.

[31] Jones S., Hruban R.H., Kamiyama M., Borges M., Zhang X., Parsons D.W., Lin J.C.-H., Palmisano E., Brune K., Jaffee E.M. (2009). Exomic sequencing identifies PALB2 as a pancreatic cancer susceptibility gene. Science.

[32] Han X., Saiyin H., Zhao J., Fang Y., Rong Y., Shi C., Lou W., Kuang T. (2017). Overexpression of miR-135b-5p promotes unfavorable clinical characteristics and poor prognosis via the repression of SFRP4 in pancreatic cancer. Oncotarget.

[33] Si L., Xu L., Yin L., Qi Y., Han X., Xu Y., Zhao Y., Liu K., Peng J. (2017). Potent effects of dioscin against pancreatic cancer via miR-149-3P-mediated inhibition of the Akt1 signalling pathway. Br. J. Pharmacol..

[34] Hu X., Wu J., Xu J. (2023). UCA1 executes an oncogenic role in pancreatic cancer by regulating miR-582-5p/BRCC3. Front. Oncol..

[35] de Visser K.E., Joyce J.A. (2023). The evolving tumor microenvironment: From cancer initiation to metastatic outgrowth. Cancer Cell.

[36] Demšar J., Curk T., Erjavec A., Gorup Č., Hočevar T., Milutinovič M., Možina M., Polajnar M., Toplak M., Starič A. (2013). Orange: Data Mining Toolbox in Python. J. Mach. Learn. Res..

[37] Chen Y., Wang X. (2022). Evaluation of efficiency prediction algorithms and development of ensemble model for CRISPR/Cas9 gRNA selection. Bioinformatics.

[38] Kang Y.J., Pan L., Liu Y., Rong Z., Liu J., Liu F. (2025). GEPIA3: Enhanced drug sensitivity and interaction network analysis for cancer research. Nucleic Acids Res..

[39] Yuan M., Zhang C., Von Feilitzen K., Zwahlen M., Shi M., Li X., Yang H., Song X., Turkez H., Uhlén M. (2024). The Human Pathology Atlas for deciphering the prognostic features of human cancers. EBioMedicine.

[40] Chandrashekar D.S., Karthikeyan S.K., Korla P.K., Patel H., Shovon A.R., Athar M., Netto G.J., Qin Z.S., Kumar S., Manne U. (2022). UALCAN: An update to the integrated cancer data analysis platform. Neoplasia.

[41] Yang S., He P., Wang J., Schetter A., Tang W., Funamizu N., Yanaga K., Uwagawa T., Satoskar A.R., Gaedcke J. (2016). A novel MIF signaling pathway drives the malignant character of pancreatic cancer by targeting NR3C2. Cancer Res..

[42] Lánczky A., Győrffy B. (2021). Web-based survival analysis tool tailored for medical research (KMplot): Development and implementation. J. Med. Internet Res..

[43] Wu H.W., Wu J.D., Yeh Y.P., Wu T.H., Chao C.-H., Wang W., Chen T.-W. (2023). DoSurvive: A webtool for investigating the prognostic power of a single or combined cancer biomarker. iScience.

[44] Cui H., Zhao G., Lu Y., Zuo S., Duan D., Luo X., Zhao H., Li J., Zeng Z., Chen Q. (2025). TIMER3: An enhanced resource for tumor immune analysis. Nucleic Acids Res..

[45] Szklarczyk D., Nastou K., Koutrouli M., Kirsch R., Mehryary F., Hachilif R., Hu D., E Peluso M., Huang Q., Fang T. (2025). The STRING database in 2025: Protein networks with directionality of regulation. Nucleic Acids Res..

[46] Szklarczyk D., Gable A.L., Lyon D., Junge A., Wyder S., Huerta-Cepas J., Simonovic M., Doncheva N.T., Morris J.H., Bork P. (2019). STRING v11: Protein-protein association networks with increased coverage, supporting functional discovery in genome-wide experimental datasets. Nucleic Acids Res..

[47] McGeary S.E., Lin K.S., Shi C.Y., Pham T.M., Bisaria N., Kelley G.M., Bartel D.P. (2019). The biochemical basis of microRNA targeting efficacy. Science.

[48] Chen Y., Wang X. (2020). miRDB: An online database for prediction of functional microRNA targets. Nucleic Acids Res..

[49] Chen G., Wang Z., Wang D., Qiu C., Liu M., Chen X., Zhang Q., Yan G., Cui Q. (2012). LncRNADisease: A database for long-non-coding RNA-associated diseases. Nucleic Acids Res..

[50] Li J.H., Liu S., Zhou H., Qu L.-H., Yang J.-H. (2013). starBase v2.0: Decoding miRNA-ceRNA, miRNA-ncRNA and protein–RNA interaction networks from large-scale CLIP-Seq data. Nucleic Acids Res..

[51] Edgar R., Domrachev M., Lash A.E. (2002). Gene Expression Omnibus: NCBI gene expression and hybridization array data repository. Nucleic Acids Res..

[52] Janky R., Binda M.M., Allemeersch J., Broeck A.V.D., Govaere O., Swinnen J.V., Roskams T., Aerts S., Topal B. (2016). Prognostic relevance of molecular subtypes and master regulators in pancreatic ductal adenocarcinoma. BMC Cancer.

[53] Kim S., Jung B.K., Kim J., Jeon J.H., Jang S.H., Kim M., Kim C.-S., Jang H. (2024). Newcastle disease virus harboring the PTEN gene inhibits pancreatic cancer growth by inhibiting PI3K/AKT/mTOR signaling and activating apoptosis. Mol. Ther. Oncol..

[54] Moffitt R.A., Marayati R., Flate E.L., Volmar K.E., Loeza S.G.H., Hoadley K.A., Rashid N.U., Williams L.A., Eaton S.C., Chung A.H. (2015). Virtual microdissection identifies distinct tumor- and stroma-specific subtypes of pancreatic ductal adenocarcinoma. Nat. Genet..

[55] Madrigal P., Thanki A.S., Fexova S., Yu I.D., Chatzigeorgiou A., Zucchi I., Calles J.C.M., Vilmovsky L., Khen A., Zhao L. (2026). Expression Atlas in 2026: Enabling FAIR and open expression data through community collaboration and integration. Nucleic Acids Res..

