# Supplementary material for: Exploratory Multi-Platform Bioinformatic Analysis of MEAK7 and Its Molecular, Survival, and Immune Associations in Pancreatic Ductal Adenocarcinoma
Source: Int J Mol Sci. 2026 Aug 25;27(17):7610. doi: 10.3390/ijms27177610 (PMC13565933; doi:10.3390/ijms27177610)
Supplement: Supplementary file 1 [file ijms-27-07610-s001.zip › Supplementary Table S3.pdf]

Supplementary Table S3

LncRNA name	Disease name	Dysfunction type	Description	Chr	Start	End	Strand	Species	Alias	Genbank	Sequence	Reference
HOTAIR	pancreatic cancer	Expression	In approximately one-quarter of human breast cancers, HOTAIR is highly induced, while its elevated levels are also predictive of metastasis and disease progression in other cancers, such as colon, colorectal, gastrointestinal, pancreatic and liver cancer.	chr12	53962308	53974956	-	Human	HOXAS; HOXC-AS4; HOXC11-AS1; NCRNA00072	NR_003716	Gene / RNA	24667321
MALAT1	pancreatic cancer	Regulation	Sequesters SR splicing factors to regulate alternative splicing.	chr11	65497679	65504494	+	Human	HCN; LINC00047; NCRNA00047; NEAT2; PRO2853	NR_002819	Gene / RNA	22996375
PVT1	pancreatic cancer	Expression	PVT1 gene is a regulator of Gemcitabine sensitivity and showed that functional inactivation of the PVT1 gene leads to enhanced Gemcitabine sensitivity in human pancreatic cancer ASPC-1 cells.	chr8	127794533	128101253	+	Human	LINC00079; MYC; NCRNA00079; onco-lncRNA-100	NR_003367	Gene / RNA	21316338
HULC	pancreatic cancer	Regulation	HULC promotes tumorigenesis by regulating multiple pathways, such as	chr6	8652209	8653846	+	Human	HCCAT1; LINC00078; NCRNA00078	NR_004855	Gene / RNA	27781386

Supplementary Table S3

			down-regulation of EEF1E1, promotion of abnormal lipid metabolism, and up-regulation of sphingosine kinase 1.									
MALAT1	pancreatic cancer	Regulation	MALAT1 is thought to regulate tumor cell viability, invasiveness and migration	chr11	65497679	65504494	+	Human	HCN; LINC00047; NCRNA00047; NEAT2; PRO2853	NR_002819	Gene / RNA	27777857
UCA1	pancreatic cancer	Regulation	Long non-coding RNA UCA1 promotes tumorigenesis in pancreatic cancer.	chr19	15828947	15836321	+	Human	CUDR; LINC00178; NCRNA00178; UCAT1; onco-lncRNA-36	NR_015379	Gene / RNA	27562722
CCDC26	pancreatic cancer	Expression	Long noncoding RNA CCDC26 as a potential predictive biomarker contributes to tumorigenesis in pancreatic cancer	chr8	129351694	129680239	-	Human	RAM	NR_130917	Gene / RNA	27470572
HOTTIP	pancreatic cancer	Expression	Dysregulation of HOTTIP is associated with various malignancies including hepatocellular carcinoma, pancreatic cancer, gastric cancer and colorectal cancer.	chr7	27200421	27207259	+	Human	HOXA-AS6; HOXA13-AS1; NCRNA00213	NR_037843	Gene / RNA	27546609
uc.345	pancreatic cancer	Regulation	Long noncoding RNA uc.345 promotes tumorigenesis of pancreatic	N/A	N/A	N/A	N/A	Human	N/A	N/A	Gene / RNA	27689400

Supplementary Table S3

			cancer by upregulation of hnRNPL expression.									
IRAIN	pancreatic cancer	Regulation	Long non-coding RNA IRAIN suppresses apoptosis and promotes proliferation by binding to LSD1 and EZH2 in pancreatic cancer	chr15	98645863	98651221	-	Human	IGF1R-AS	NR_126453	Gene / RNA	27644252
MALAT1	pancreatic cancer	Regulation	Long Noncoding RNA MALAT1 Promotes Aggressive Pancreatic Cancer Proliferation and Metastasis via the Stimulation of Autophagy.	chr11	65497679	65504494	+	Human	HCN; LINC00047; NCRNA00047; NEAT2; PRO2853	NR_002819	Gene / RNA	27371730
HMlincRNA717	pancreatic cancer	Expression	HMlincRNA717 could be a potential prognostic biomarker for pancreatic cancer progression.	N/A	N/A	N/A	N/A	Human	N/A	N/A	Gene / RNA	27338046
LINC00673	pancreatic cancer	Regulation	A long intergenic noncoding RNA (lincRNA), LINC00673, is a potential tumor suppressor whose germline variation is associated with pancreatic cancer risk.	chr17	72403322	72592804	-	Human	HI-LNC75; HILNC75; LUCAIR1; SLNCR; SLNCR1	NR_036488	Gene / RNA	27213290
HOTAIR	pancreatic cancer	Expression	Salivary HOTAIR and PVT1 show potential as	chr12	53962308	53974956	-	Human	HOXAS; HOXC-AS4; HOXC11-AS1;	NR_003716	Gene / RNA	27028998

Supplementary Table S3

			novel non-invasive biomarkers for detecting PC.						NCRNA00072			
PVT1	pancreatic cancer	Expression	Salivary HOTAIR and PVT1 show potential as novel non-invasive biomarkers for detecting PC.	chr8	127794533	128101253	+	Human	LINC00079; MYC; NCRNA00079; onco-lncRNA-100	NR_003367	Gene / RNA	27028998
LINC-ROR	pancreatic cancer	Regulation	linc-ROR acts as an important regulator of ZEB1, can promote invasion and metastasis in PC, and may represent a novel therapeutic target.	chr18	57054571	57072119	-	Human	ROR; lincRNA-RoR	NR_048536	Gene / RNA	26898939
MEG3	pancreatic cancer	Regulation	fenofibrate inhibited pancreatic cancer cell proliferation via activation of p53 mediated by upregulation of MEG3.	chr14	100826108	100861026	+	Human	FP504; GTL2; LINC00023; NCRNA00023; PRO0518; PRO2160; onco-lncRNA-83; prebp1	NR_046472	Gene / RNA	26850851
MALAT1	pancreatic cancer	Regulation	EZH2 promotes cell migration and invasion but does not alter cell proliferation by suppressing E-cadherin, partly through association with MALAT-1 in pancreatic cancer.	chr11	65497679	65504494	+	Human	HCN; LINC00047; NCRNA00047; NEAT2; PRO2853	NR_002819	Gene / RNA	26848980
NUTF2P3	pancreatic cancer	Expression	lncRNA-NUTF2P3-001 and miR-3923 can be applied as	chr9	77580245	77580628	+	Human	NUTF2P3	ENST00000448540	Gene / RNA	26755660

Supplementary Table S3

			novel predictors and therapeutic targets for pancreatic cancer.									
LINC-ROR	pancreatic cancer	Expression	ROR is a potential novel prognostic marker to predict the clinical outcome of pancreatic cancer patients after surgery and may be a rational target for therapy.	chr 18	57054571	57072119	-	Human	ROR; lincRNA-RoR	NR_048536	Gene / RNA	26636540
ATB	pancreatic cancer	Regulation	LncRNA-ATB may play a critical role in pancreatic cancer progression and prognosis and may serve as a potential prognostic biomarker in pancreatic cancer patients.	N/A	N/A	N/A	N/A	Human	N/A	N/A	Gene / RNA	26482611
HOTTIP	pancreatic cancer	Expression	Increased HOTTIP-005 and RP11-567G11.1 expression were poor prognostic factors for patients with PC	chr 7	27200421	27207259	+	Human	HOXA-AS6; HOXA13-AS1; NCRNA00213	NR_037843	Gene / RNA	26447755
RP11-567G11.1	pancreatic cancer	Expression	Increased HOTTIP-005 and RP11-567G11.1 expression were poor prognostic factors for patients with PC	chr 3	187448845	187449450	+	Human	LINC02041	ENST00000440726	Gene / RNA	26447755

Supplementary Table S3

AF339813	pancreatic cancer	Regulation	NUF2 is consistently overexpressed in human PC and NUF2 is closely linked with human PC progression through the mediator LncRNA AF339813	N/A	N/A	N/A	N/A	Human	N/A	N/A	Gene / RNA	26045769
HOTTIP	pancreatic cancer	Regulation	The long non-coding RNA HOTTIP enhances pancreatic cancer cell proliferation, survival and migration.	chr7	27200421	27207259	+	Human	HOXA-AS6; HOXA13-AS1; NCRNA00213	NR_037843	Gene / RNA	25912306
HOTAIR	pancreatic cancer	Regulation	LncRNA HOTAIR regulates strikingly different sets of genes in Panc1 cells and in pancreatic tumors.	chr12	53962308	53974956	-	Human	HOXAS; HOXC-AS4; HOXC11-AS1; NCRNA00072	NR_003716	Gene / RNA	25912306
LINC00261	pancreatic cancer	N/A	It plays an important role in pancreatic cancer	chr20	22560553	22578642	-	Human	ALIEN; C20orf56; DEANR1; HCCDR1; NCRNA00261; onco-lncRNA-17	NR_001558	Gene / RNA	25910082
CYTOR	pancreatic cancer	N/A	It plays an important role in pancreatic cancer	chr2	87454555	87521518	+	Human	C2orf59; LINC00152; NCRNA00152	NR_024204	Gene / RNA	25910082
HNF1A-AS1	pancreatic cancer	N/A	It plays an important role in pancreatic cancer	chr12	120969838	120972292	-	Human	C12orf27; NCRNA00262	NR_024345	Gene / RNA	25910082
AFAP1-AS1	pancreatic cancer	N/A	It plays an important role in pancreatic cancer	chr4	7754090	7778928	+	Human	AFAP1-AS; AFAP1AS	NR_026892	Gene / RNA	25910082
HOTTIP	pancreatic cancer	Regulation	HOTTIP promotes cell	chr7	27200421	27207259	+	Human	HOXA-AS6; HOXA13-	NR_037843	Gene / RNA	25889214

Supplementary Table S3

			proliferation, invasion, and chemoresistance by modulating HOXA13						AS1; NCRNA00213			
MALAT1	pancreatic cancer	Regulation	MALAT-1 could promote tumorigenicity of pancreatic cancer cells in vivo	chr 11	65497679	65504494	+	Human	HCN; LINC00047; NCRNA00047; NEAT2; PRO2853	NR_002819	Gene / RNA	25811929
PVT1	pancreatic cancer	Expression	The increased expression of lncRNA PVT1 in PDAC was correlated with tumor progression, and PVT1 might be a potential molecular biomarker for predicting the prognosis of patients with PDAC.	chr 8	127794533	128101253	+	Human	LINC00079; MYC; NCRNA00079; onco-lncRNA-100	NR_003367	Gene / RNA	25668599
MALAT1	pancreatic cancer	Expression	Overexpression of long non-coding RNA MALAT1 is correlated with clinical progression and unfavorable prognosis in pancreatic cancer.	chr 11	65497679	65504494	+	Human	HCN; LINC00047; NCRNA00047; NEAT2; PRO2853	NR_002819	Gene / RNA	25481511
HULC	pancreatic cancer	Expression	Long noncoding RNA HULC is a novel biomarker of poor prognosis in patients with pancreatic cancer	chr 6	8652209	8653846	+	Human	HCCAT1; LINC00078; NCRNA00078	NR_004855	Gene / RNA	25412939
MALAT1	pancreatic cancer	Regulation	Elevated expression level of long noncoding RNA	chr 11	65497679	65504494	+	Human	HCN; LINC00047; NCRNA00047;	NR_002819	Gene / RNA	25269958

Supplementary Table S3

			MALAT-1 facilitates cell growth, migration and invasion in pancreatic cancer						NEAT2; PRO2853			
H19	pancreatic cancer	Regulation	H19 promotes pancreatic cancer metastasis by derepressing let-7's suppression of its target HMGA2-mediated EMT	chr11	1995176	2001466	-	Human	ASM; ASM1; BWS; D11S813E; LINC00008; NCRNA00008; WT2	NR_131223	Gene / RNA	24920070
GAS5	pancreatic cancer	Regulation	Downregulation of gas5 increases pancreatic cancer cell proliferation by regulating CDK6	chr1	173863901	173867987	-	Human	NCRNA00030; SNHG2	NR_002578	Gene / RNA	24026436
HOTAIR	pancreatic cancer	Regulation	HOTAIR is a negative prognostic factor and exhibits pro-oncogenic activity in pancreatic cancer	chr12	53962308	53974956	-	Human	HOXAS; HOXC-AS4; HOXC11-AS1; NCRNA00072	NR_003716	Gene / RNA	22614017
