# Supplementary material for: Exploratory Multi-Platform Bioinformatic Analysis of MEAK7 and Its Molecular, Survival, and Immune Associations in Pancreatic Ductal Adenocarcinoma
Source: Int J Mol Sci. 2026 Aug 25;27(17):7610. doi: 10.3390/ijms27177610 (PMC13565933; doi:10.3390/ijms27177610)
Supplement: Supplementary file 1 [file ijms-27-07610-s001.zip › Supplementary Methods and Results for Exploratory Machine.pdf]

Supplementary Methods and Results for Exploratory Machine-Learning, CRISPR/Cas9, and Drug-Sensitivity Analyses

1. Supplementary Methods

1.1. Exploratory Machine-Learning-Based Tissue Classification Using a MEAK7-Centered Gene Panel

The same normalized and batch-corrected GSE62452 expression matrix described in Section 4.2 was used for the machine-learning analysis. The cohort comprised 69 PDAC tumor and 61 adjacent non-tumor pancreatic tissue samples, including 60 matched tumor-adjacent non-tumor pairs and 10 unmatched samples. The predictor panel was defined before model evaluation based on the STRING functional association network and consisted of MEAK7 and ten genes showing high-confidence functional associations with MEAK7 (combined score >0.9): ATP6V1A, ATP6V1G1, ATP6V1C1, ATP6AP1, ATP6AP2, ATP6V0C, ATP6V0E1, ATP6V1D, ATP6V1H, and RNASEK. All 11 prespecified genes were retained, and no outcome-guided feature selection was performed.

Machine-learning analyses were performed using Orange Data Mining software, version 3.40.0 [1]. Tissue group was defined as the categorical target variable, with tumor tissue designated as the positive class and adjacent non-tumor tissue as the negative class. Sample_ID, Pair_ID, and CV_Fold were defined as metadata and were not included as predictor features. Model performance was evaluated using fixed, pair-aware five-fold cross-validation. The predefined categorical CV_Fold meta-variable was used to define the folds through the "Cross validation by feature" option in the Test and Score widget. Tumor and adjacent non-tumor samples sharing the same Pair_ID were assigned to the same fold. Each fold comprised 12 matched pairs and two unmatched samples, resulting in 26 samples per fold. Accordingly, in each iteration, 104 samples were used for training and 26 samples were held out for testing; each sample appeared in the test partition exactly once.

Gene-expression features were standardized to a mean of 0 and a variance of 1. The Preprocess widget was connected to the Preprocessor input of Test and Score so that standardization parameters were estimated separately within each training fold and subsequently applied to the corresponding held-out fold. This procedure represented feature scaling for scale-sensitive machine-learning algorithms rather than renormalization of the microarray expression data and prevented information from the test folds from entering the model-training process.

Seven classification algorithms were compared: logistic regression with L2 regularization (C=1); Random Forest with 500 trees; scikit-learn Gradient Boosting with 100 trees, a learning rate of 0.1, and a maximum tree depth of 3; C-SVM with a radial basis function kernel (C=1 and gamma set to automatic); decision tree with a minimum of five samples per leaf, a minimum of ten samples required for splitting, and a maximum depth of 4; k-nearest neighbors with five neighbors, Euclidean distance, and uniform weighting; and an artificial neural network with one hidden layer containing five neurons, tanh activation, the L-BFGS-B solver, an L2

regularization parameter of α=0.01, and a maximum of 1,000 iterations. The replicable training option was enabled for algorithms with stochastic components, where available. Other parameters were retained at their Orange default settings, and no automated or post hoc hyperparameter optimization based on model-performance results was performed.

Performance was assessed using the area under the receiver operating characteristic curve (AUC), classification accuracy (CA), F1 score, precision, sensitivity, specificity, and Matthews correlation coefficient (MCC), with tumor tissue defined as the positive target class. Performance measures, ROC curves, and confusion matrices were derived from the aggregated out-of-fold predictions obtained across the five fixed folds. A graphical representation of the Orange Data Mining analysis workflow is provided as Figure S1 in scalable vector graphics format. Because model development and internal evaluation were conducted within the same processed microarray cohort, the results were interpreted as exploratory internal-validation findings rather than externally validated clinical diagnostic performance.

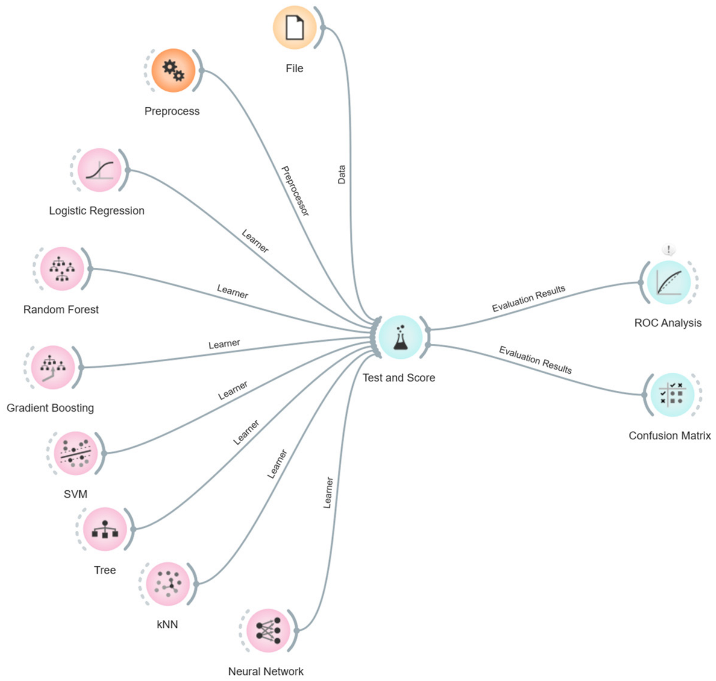

Figure S1. Orange-based machine-learning workflow for exploratory classification of PDAC tumor and adjacent non-tumor tissues. Schematic representation of the machine-learning workflow implemented in Orange for the exploratory classification of PDAC tumor and adjacent non-tumor pancreatic tissues using the MEAK7-centered gene panel. Following data import and preprocessing, seven classification algorithms—logistic regression, random forest, gradient boosting, support vector machine (SVM), decision tree, k-nearest neighbors (kNN), and neural network—were evaluated using the Test and Score module.

1.2. In Silico Prioritization of CRISPR/Cas9 gRNAs Targeting MEAK7

Potential MEAK7-directed guide RNA (gRNA) candidates were screened in silico using CRISPRdb (https://crisprdb.org/cgi-bin/search_gRNA.cgi; accessed June 16, 2026). Only guide candidates compatible with the SpCas9-associated NGG protospacer-adjacent motif (PAM) were considered. Candidate guides were ranked by integrating CRISPRdb-derived efficiency scores with the available off-target annotations, and 20 guides with favorable overall in silico profiles were retained for reporting. Available sequence-level parameters, such as GC content, were also considered during the candidate review. This analysis aimed to generate a preliminary list of MEAK7-directed gRNA candidates for future experimental perturbation studies. CRISPR guide RNAs targeting MEAK7 were obtained from the CRISPRdb platform. The analysis was based on the human reference genome and the guide RNA design pipeline implemented by the platform. On-target gRNA efficacy was assessed using the CRISPRdb ensemble_ridge model, which integrates predictions from five established algorithms—CRISPRon, DeepSpCas9, DeepHF, sgDesigner, and TSAM—through ridge regression. Scores range from 50 to 100, with higher values indicating greater predicted editing activity; these values do not represent experimentally measured editing efficiencies. CRISPRdb directly provided the candidate gRNA sequences and platform-reported on-target activity scores. Potential off-targets were screened using two CRISPRdb-defined criteria: an exact 13-nucleotide seed-sequence match or at least 85% overall sequence homology to the gRNA based on BLAST alignment. The off-target search was restricted to known exon regions of protein-coding and non-coding genes [2]. Transcript-level annotation, exon assignment, and chromosome-based genomic coordinates and predicted off-target loci were not available in the accessible CRISPRdb output and were therefore not reported.

1.3. Drug Sensitivity Analysis Associated with MEAK7 Expression

To evaluate the possible relationships between *MEAK7* expression and drug sensitivity, the drug sensitivity module of the Gene Set Cancer Analysis (GSCA) platform (https://guolab.wchscu.cn/GSCA/#/drug; access date: 18 June 2026) was used. The analysis was based on examining the correlations between *MEAK7* expression levels and drug response profiles in the Genomics of Drug Sensitivity in Cancer (GDSC) and Cancer Therapeutics Response Portal (CTRP) datasets. The relationship between *MEAK7* expression and drug response was evaluated using correlation coefficients and false discovery rate (FDR) values. An FDR $\leq$ 0.05 was considered statistically significant. The results were visualized using a color scale indicating the direction and strength of the correlation and dot sizes reflecting the level of statistical significance. This analysis was conducted as an exploratory in silico pharmacogenomic assessment to investigate whether *MEAK7* expression is associated with sensitivity or resistance to different anticancer agents.

Drug sensitivity analyses were performed using the GSCA platform, which integrates pan-cancer pharmacogenomic data from GDSC and CTRP, encompassing diverse solid-tumor and hematologic cancer-cell-

line lineages. According to the GSCA analytical framework, the integrated datasets comprise 1001 cancer cell lines and 251 compounds from GDSC, and 960 cancer cell lines and 481 compounds from CTRP. Drug sensitivity was evaluated by correlating gene expression with drug IC50 values using Pearson correlation analysis, and statistical significance was assessed after false discovery rate (FDR) correction.

2. Supplementary Results

2.1. Exploratory Machine-Learning-Based Tissue Classification Using a MEAK7-Centered Gene Panel

Utilizing the STRING-derived functional association network, an 11-gene panel, including *MEAK7* and ten related genes (*ATP6V1A, ATP6V1G1, ATP6V1C1, ATP6AP1, ATP6AP2, ATP6V0C, ATP6V0E1, ATP6V1D, ATP6V1H,* and *RNASEK*), was analyzed for the exploratory classification of PDAC tumors and adjacent non-tumor pancreatic tissues. The model's performance was evaluated through pair-aware five-fold cross-validation, ensuring that matched tumor and adjacent non-tumor samples were allocated to the same fold.

Among the seven models assessed, Support Vector Machine (SVM) and logistic regression achieved the highest AUC values, measuring 0.837 and 0.836, respectively (Table S5). SVM demonstrated marginally superior accuracy, precision, specificity, and Matthews Correlation Coefficient (MCC), while logistic regression exhibited slightly enhanced sensitivity and F1 score. SVM accurately classified 99 out of 130 samples, with true negatives (TN) at 47, true positives (TP) at 52, false positives (FP) at 14, and false negatives (FN) at 17. In contrast, logistic regression accurately classified 98 samples, with TN at 44, TP at 54, FP at 17, and FN at 15. Gradient Boosting and Neural Network models displayed relatively lower internal classification performance, particularly concerning classification accuracy and MCC.

Table S5. Preliminary internal classification performance of machine-learning models based on MEAK7-associated gene-expression features.

Model	AUC	CA	F1	Precision	Sensitivity	Specificity	MCC
SVM	0.837	0.762	0.770	0.788	0.754	0.770	0.523
Logistic Regression	0.836	0.754	0.771	0.761	0.783	0.721	0.505
Random Forest	0.797	0.715	0.726	0.742	0.710	0.721	0.431
kNN	0.775	0.738	0.754	0.754	0.754	0.721	0.475
Neural Network	0.740	0.646	0.689	0.646	0.739	0.541	0.286
Tree	0.727	0.708	0.708	0.754	0.667	0.754	0.421
Gradient Boosting	0.687	0.638	0.671	0.649	0.696	0.574	0.272

AUC, area under the receiver operating characteristic curve; CA, classification accuracy; F1, F1 score; MCC, Matthews correlation coefficient; kNN, k-nearest neighbors; SVM, support vector machine. Tumor tissue was defined as the positive class. Values represent performance estimates obtained from out-of-fold predictions aggregated across the five fixed folds.

2.2 ***Computational Assessment of MEAK7 for CRISPR/Cas9-Based Targeting***

To explore the suitability of *MEAK7* for CRISPR/Cas9-based perturbation, 20 putative guide RNA sequences were screened in silico. The in silico activity estimates indicated favorable predicted performance, with scores spanning 81.0–99.3. Among the 20 candidate gRNAs, 11 showed no computationally predicted off-target sites, whereas nine were flagged as having one or more potential off-target sites according to the prediction criteria implemented by the CRISPRdb platform. This finding represents an in silico prediction and does not exclude the possibility of off-target effects under experimental conditions. These results also highlight the need to balance predicted on-target activity against possible off-target liability during final guide selection. Overall, the screening identified several MEAK7-directed guide candidates with favorable predicted activity profiles and limited off-target concern (Figure S2). No multiple-testing correction was applied to the CRISPR guide-RNA screening because this analysis did not involve a family of hypothesis tests or p-value-based statistical comparisons. Candidate guides were evaluated descriptively according to platform-derived predicted on-target activity scores and computational off-target annotations.

Rank	gRNA Sequence (5' → 3')	Potency Score (0–100)	Off-Target Status
1	CCTGTTGAAAGGAAACTCCG	99.3	No off-target identified
2	CAGTCATAGTCCAGCCACTG	95.8	No off-target identified
3	CGTTGATGTACATGACAGAG	94.6	No off-target identified
4	CAGCCTGGTGACCATCTCTG	93.2	No off-target identified
5	GAGGGTCGACCTGACAGGGA	91.6	No off-target identified
6	ATGGTCACCAGGCTGTATGA	91.3	No off-target identified
7	ACATGACAGAGAGGACATCC	88.2	No off-target identified
8	GCTGAGAGGCTGGACTGGGA	86.3	No off-target identified
9	CCAGGCTGTATGATGGCATG	86.0	No off-target identified
10	GTTTGCCTCTTGCTCTTGGG	83.7	No off-target identified
11	CATGACAAGCATGTGTTCGG	82.4	No off-target identified
12	AGCCACAGACAGGAGCTGAG	99.0	Potential off-targets identified
13	TGTGGCCACATCACTCACCG	99.0	Potential off-targets identified
14	GGAGCTCAGACGAAAAGAGC	93.1	Potential off-targets identified
15	TGGACTGGGAAGGAAGCCCC	92.1	Potential off-targets identified
16	ACGAAAAGAGCAGGCACCAG	86.3	Potential off-targets identified
17	TCAGATCAAGAGACGAGCAC	83.3	Potential off-targets identified
18	TGTGGCCATATTCCTGAGTG	83.3	Potential off-targets identified
19	AGGGTCCCCGGTGAGTGATG	81.0	Potential off-targets identified
20	GGAAGCTCTTCCCCCAGAGA	81.0	Potential off-targets identified

No off-target identified: No significant off-target sites predicted by the computational analysis.

Potential off-targets identified: One or more potential off-target sites predicted by the computational analysis.

Potency Score (0–100): Higher scores indicate higher predicted on-target editing efficiency.

Note: *Off-target status represents computational prediction only and does not constitute experimental validation.*

Figure S2. Candidate gRNAs targeting MEAK7 ranked according to predicted on-target potency and computational off-target assessment. Potency score denotes an in silico estimate of the expected on-target editing activity of each guide RNA, with larger values reflecting more favorable predicted performance. Off-target annotation was assigned after screening each candidate guide for possible, unintended genomic alignments. For U6 promoter-driven guide RNA expression, a 5′ guanine is commonly favored for efficient transcription initiation; therefore, guides lacking an initial G may be modified by adding a single guanine at the 5′ end before the construct design. Each guide sequence included in the figure was selected as a MEAK7-directed candidate.

2.3 *Drug Sensitivity Associations*

The relationship between *MEAK7* mRNA expression and drug response profiles was assessed using the drug sensitivity module of the Gene Set Cancer Analysis (GSCA) platform. The Genomics of Drug Sensitivity in Cancer (GDSC) and Cancer Therapeutics Response Portal (CTRP) datasets were used for the analyses. GDSC analysis revealed significant correlations between *MEAK7* mRNA expression and

sensitivity profiles of multiple pharmacological agents. The presence of both positive and negative correlations suggests that the relationship between *MEAK7* expression and predicted drug response may exhibit agent-specific differences. The CTRP analysis also revealed significant associations between *MEAK7* expression and drug sensitivity. Overall, these findings indicate that *MEAK7* expression may be associated with pharmacogenomic response patterns in a dataset-dependent manner (Figure S3). To enhance mechanistic interpretation, all compounds significantly correlated with *MEAK7* expression were categorized based on their primary pharmacological mechanisms, as detailed in Supplementary Table S1. Within the GDSC dataset, positive correlations were predominantly observed for epigenetic modulators, such as AR-42, belinostat, CAY10603, I-BET-762, tubastatin A, UNC0638, and vorinostat, as well as inhibitors of cell-cycle, PI3K/PDK1, JAK/STAT, NF-κB, and apoptosis-related pathways. These correlations suggest a potential association between elevated MEAK7 expression and reduced drug sensitivity. Conversely, negative correlations were identified for afatinib, bleomycin, docetaxel, and gefitinib, indicating that increased MEAK7 expression may correlate with heightened sensitivity to these agents. In the CTRP dataset, all significantly associated compounds demonstrated positive correlations with MEAK7 expression, encompassing HDAC and BET inhibitors, cell-cycle kinase inhibitors, conventional cytotoxic agents, redox-modulating compounds, and microtubule-targeting agents.

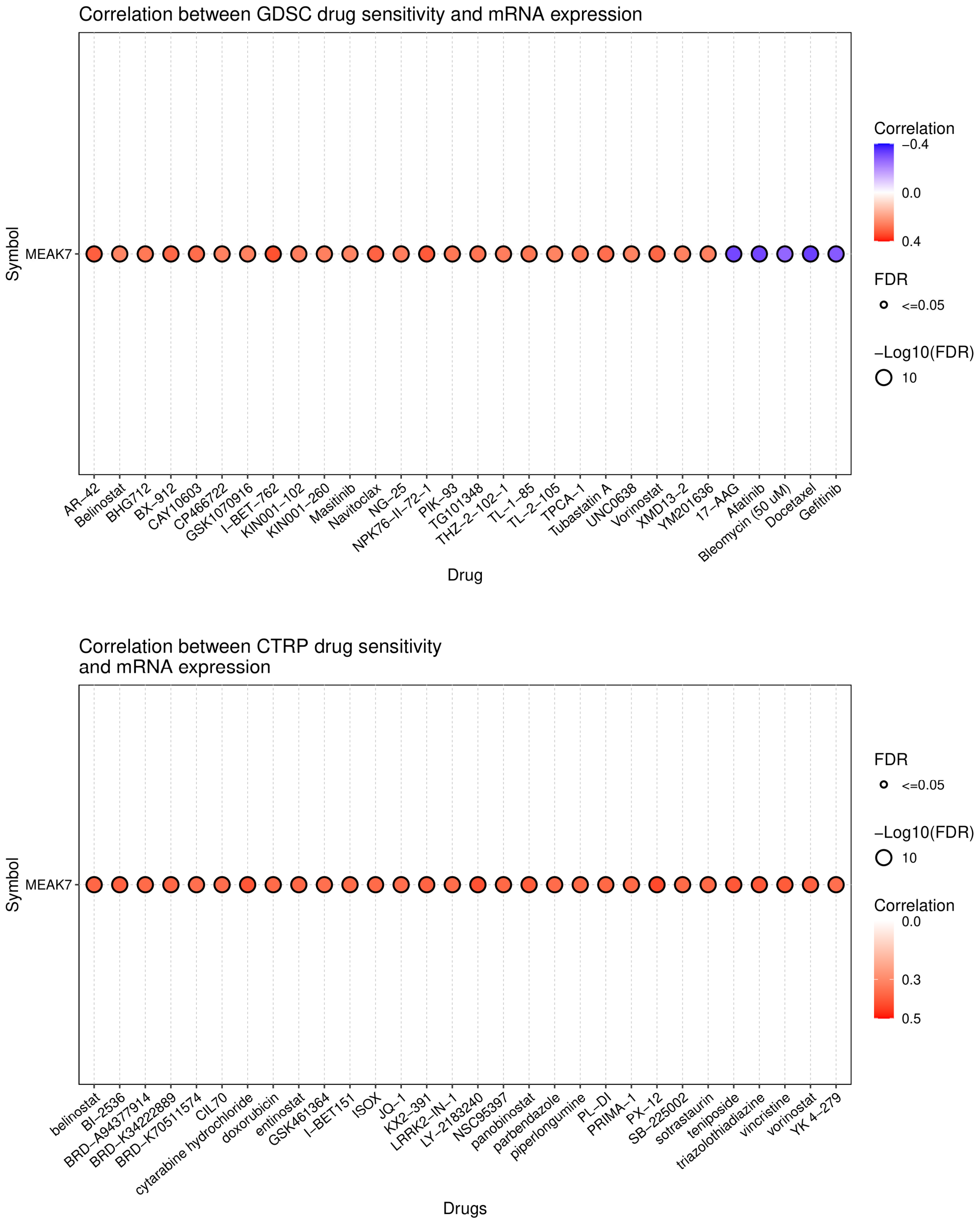

Figure S3. GSCA-based analysis of the association between *MEAK7* mRNA expression and drug sensitivity in the GDSC and CTRP datasets. The upper panel shows the correlation between *MEAK7* mRNA expression and drug sensitivity profiles in the Genomics of Drug Sensitivity in Cancer (GDSC) dataset, whereas the lower panel presents the corresponding analysis in the Cancer Therapeutics Response Portal (CTRP) dataset. The dot color represents the direction and magnitude of the correlation, and the dot size indicates −log10(FDR). Significant associations were defined as FDR ≤ 0.05. Positive correlations may indicate an association with drug resistance, whereas negative correlations may indicate an association with increased drug sensitivity.

3. Supplementary Discussion

In the exploratory machine-learning analysis based on a MEAK7-centered gene panel derived from the STRING functional association network, logistic regression and SVM showed the highest classification performance among the evaluated models. These findings suggest that the panel may capture expression differences between PDAC tumor and adjacent non-tumor pancreatic tissues. However, because model development and evaluation were conducted within a single processed microarray cohort, the resulting estimates may remain cohort- and platform-specific despite the use of pair-aware cross-validation. These findings should therefore be regarded as exploratory and require validation in an independent cohort before any clinical interpretation.

The CRISPRdb-based screening identified several MEAK7-directed gRNA candidates with favorable predicted on-target activity and limited computational off-target annotations. However, these findings are entirely prediction-based and do not establish editing efficiency, genomic specificity, or functional consequences in PDAC models. Experimental validation of on-target editing, potential off-target effects, and resulting cellular phenotypes will therefore be required before these candidates can be considered for functional perturbation studies.

Qualitative mechanistic grouping showed that epigenetic modulators, particularly HDAC and BET inhibitors, were frequently represented among compounds positively correlated with MEAK7 expression. This descriptive pattern suggests a potential association between MEAK7 expression and response to chromatin-targeting agents; however, it does not establish a distinct epigenetic state or a causal drug-resistance mechanism. In contrast, the negative correlations identified for afatinib, gefitinib, docetaxel, and bleomycin in the GDSC dataset indicate potential vulnerabilities to EGFR-targeted and conventional cytotoxic therapies. The discordant directions observed for certain pharmacological classes between the GDSC and CTRP datasets, along with the exclusively positive correlations in CTRP, may be attributed to variations in cell-line composition, drug-response metrics, dose ranges, and screening methodologies. Consequently, these associations should be considered hypothesis-generating and warrant experimental validation in pancreatic cancer models.

Supplementary References

[1] Demšar J, Curk T, Erjavec A, et al. Orange: Data Mining Toolbox in Python. Journal of Machine Learning Research [Internet]. 2013 [cited 2026 Aug 2];14(71):2349–2353.

[2] Chen Y, Wang X. Evaluation of efficiency prediction algorithms and development of ensemble model for CRISPR/Cas9 gRNA selection. Bioinformatics [Internet]. 2022 [cited 2026 Aug 3];38(23):5175–5181.
