# Supplementary material for: Exploratory Multi-Platform Bioinformatic Analysis of MEAK7 and Its Molecular, Survival, and Immune Associations in Pancreatic Ductal Adenocarcinoma
Source: Int J Mol Sci. 2026 Aug 25;27(17):7610. doi: 10.3390/ijms27177610 (PMC13565933; doi:10.3390/ijms27177610)
Supplement: Supplementary file 1 [file ijms-27-07610-s001.zip › Supplementary Table S1.pdf]

Supplementary Table S1

Database	Mechanistic class	Drug	Principal target/mechanism	Correlation with MEAK7	Drug-response interpretation
GDSC	Epigenetic modulator	AR-42	Pan-HDAC inhibitor	Positive	Potentially lower sensitivity
GDSC	Epigenetic modulator	Belinostat	Pan-HDAC inhibitor	Positive	Potentially lower sensitivity
GDSC	Receptor tyrosine kinase inhibitor	BHG712	EphB4 kinase inhibitor	Positive	Potentially lower sensitivity
GDSC	PI3K/PDK1 pathway inhibitor	BX-912	PDK1 inhibitor	Positive	Potentially lower sensitivity
GDSC	Epigenetic modulator	CAY10603	HDAC6-selective inhibitor	Positive	Potentially lower sensitivity
GDSC	DNA-damage response inhibitor	CP466722	ATM kinase inhibitor	Positive	Potentially lower sensitivity
GDSC	Cell-cycle kinase inhibitor	GSK1070916	Aurora B/Aurora C inhibitor	Positive	Potentially lower sensitivity
GDSC	Epigenetic modulator	I-BET-762	BET bromodomain inhibitor	Positive	Potentially lower sensitivity
GDSC	Experimental kinase inhibitor	KIN001-102	Broad/experimental kinase inhibitor	Positive	Potentially lower sensitivity
GDSC	Experimental kinase inhibitor	KIN001-260	Experimental kinase inhibitor	Positive	Potentially lower sensitivity
GDSC	Receptor tyrosine kinase inhibitor	Masitinib	KIT, PDGFR and related kinases	Positive	Potentially lower sensitivity
GDSC	Apoptosis regulator	Navitoclax	BCL-2/BCL-XL/BCL-W inhibitor	Positive	Potentially lower sensitivity

Supplementary Table S1

GDSC	MAPK pathway inhibitor	NG-25	TAK1/MAP4K2-related kinase inhibitor	Positive	Potentially lower sensitivity
GDSC	Experimental kinase inhibitor	NPK76-II-72-1	Experimental multikinase inhibitor	Positive	Potentially lower sensitivity
GDSC	PI3K pathway inhibitor	PIK-93	PI4K/PI3K-family inhibitor	Positive	Potentially lower sensitivity
GDSC	JAK/STAT pathway inhibitor	TG101348	JAK2 inhibitor; fedratinib	Positive	Potentially lower sensitivity
GDSC	Transcriptional CDK inhibitor	THZ-2-102-1	Covalent CDK7-related inhibitor	Positive	Potentially lower sensitivity
GDSC	Experimental kinase inhibitor	TL-1-85	Experimental kinase-directed compound	Positive	Potentially lower sensitivity
GDSC	Experimental kinase inhibitor	TL-2-105	Experimental kinase-directed compound	Positive	Potentially lower sensitivity
GDSC	NF-κB pathway inhibitor	TPCA-1	IKKβ inhibitor	Positive	Potentially lower sensitivity
GDSC	Epigenetic modulator	Tubastatin A	HDAC6-selective inhibitor	Positive	Potentially lower sensitivity
GDSC	Epigenetic modulator	UNC0638	G9a/GLP histone methyltransferase inhibitor	Positive	Potentially lower sensitivity
GDSC	Epigenetic modulator	Vorinostat	Pan-HDAC inhibitor	Positive	Potentially lower sensitivity
GDSC	MAPK/kinase inhibitor	XMD13-2	Experimental kinase inhibitor	Positive	Potentially lower sensitivity
GDSC	Vesicular trafficking/lipid kinase inhibitor	YM201636	PIKfyve inhibitor	Positive	Potentially lower sensitivity

Supplementary Table S1

GDSC	Proteostasis inhibitor	17-AAG	HSP90 inhibitor	Positive	Potentially lower sensitivity
GDSC	EGFR-family targeted therapy	Afatinib	Irreversible EGFR/HER2/HER4 inhibitor	Negative	Potentially higher sensitivity
GDSC	DNA-damaging chemotherapy	Bleomycin (50 μM)	DNA strand-break-inducing agent	Negative	Potentially higher sensitivity
GDSC	Microtubule-targeting chemotherapy	Docetaxel	Microtubule stabilizer	Negative	Potentially higher sensitivity
GDSC	EGFR-targeted therapy	Gefitinib	EGFR tyrosine kinase inhibitor	Negative	Potentially higher sensitivity
CTRP	Epigenetic modulator	Belinostat	Pan-HDAC inhibitor	Positive	Potentially lower sensitivity
CTRP	Cell-cycle kinase inhibitor	BI-2536	PLK1 inhibitor; also reported BRD4 activity	Positive	Potentially lower sensitivity
CTRP	Experimental compound	BRD-A94377914	Broad Institute annotated screening compound	Positive	Potentially lower sensitivity
CTRP	Experimental compound	BRD-K34222889	Broad Institute annotated screening compound	Positive	Potentially lower sensitivity
CTRP	Experimental compound	BRD-K70511574	Broad Institute annotated screening compound	Positive	Potentially lower sensitivity
CTRP	Experimental cytotoxic compound	CIL70	Experimental cytotoxic small molecule	Positive	Potentially lower sensitivity
CTRP	Antimetabolite chemotherapy	Cytarabine hydrochloride	DNA polymerase inhibition after incorporation	Positive	Potentially lower sensitivity

Supplementary Table S1

CTRP	DNA-damaging chemotherapy	Doxorubicin	Topoisomerase II inhibition and free-radical damage	Positive	Potentially lower sensitivity
CTRP	Epigenetic modulator	Entinostat	Class I HDAC inhibitor	Positive	Potentially lower sensitivity
CTRP	Cell-cycle kinase inhibitor	GSK461364	PLK1 inhibitor	Positive	Potentially lower sensitivity
CTRP	Epigenetic modulator	I-BET151	BET bromodomain inhibitor	Positive	Potentially lower sensitivity
CTRP	Epigenetic modulator	ISOX	HDAC6-directed/epigenetic probe	Positive	Potentially lower sensitivity
CTRP	Epigenetic modulator	JQ-1	BET bromodomain inhibitor	Positive	Potentially lower sensitivity
CTRP	Src/cytoskeletal kinase inhibitor	KX2-391	Src and tubulin-polymerization inhibitor	Positive	Potentially lower sensitivity
CTRP	Kinase inhibitor	LRRK2-IN-1	LRRK2 inhibitor	Positive	Potentially lower sensitivity
CTRP	Endocannabinoid-metabolism modulator	LY-2183240	FAAH/endocannabinoid uptake inhibitor	Positive	Potentially lower sensitivity
CTRP	Protein-tyrosine phosphatase inhibitor	NSC95397	CDC25 dual-specificity phosphatase inhibitor	Positive	Potentially lower sensitivity
CTRP	Epigenetic modulator	Panobinostat	Pan-HDAC inhibitor	Positive	Potentially lower sensitivity
CTRP	Microtubule-targeting agent	Parbendazole	Benzimidazole; microtubule disruption	Positive	Potentially lower sensitivity

Supplementary Table S1

CTRP	Oxidative-stress/apoptosis modulator	Piperlongumine	ROS-inducing electrophilic compound	Positive	Potentially lower sensitivity
CTRP	Experimental compound	PL-DI	Experimental targeted screening compound	Positive	Potentially lower sensitivity
CTRP	p53-reactivating compound	PRIMA-1	Mutant-p53 reactivation/redox modulation	Positive	Potentially lower sensitivity
CTRP	Redox regulator	PX-12	Thioredoxin-1 inhibitor	Positive	Potentially lower sensitivity
CTRP	Chemokine receptor antagonist	SB-225002	CXCR2 antagonist	Positive	Potentially lower sensitivity
CTRP	Steroidal alkaloid	Solasodine	Experimental natural-product cytotoxic agent	Positive	Potentially lower sensitivity
CTRP	DNA-damaging chemotherapy	Teniposide	Topoisomerase II inhibitor	Positive	Potentially lower sensitivity
CTRP	Microtubule-targeting agent	Thiazolovindrazine	Tubulin/microtubule-disrupting agent	Positive	Potentially lower sensitivity
CTRP	Microtubule-targeting chemotherapy	Vincristine	Vinca alkaloid; microtubule depolymerization	Positive	Potentially lower sensitivity
CTRP	Epigenetic modulator	Vorinostat	Pan-HDAC inhibitor	Positive	Potentially lower sensitivity
CTRP	Transcription-factor/fusion-protein inhibitor	YK-4-279	EWS–FLI1/RNA-helicase-associated inhibitor	Positive	Potentially lower sensitivity

Note: Positive correlations indicate that higher MEAK7 expression is associated with higher drug-response values and thus potentially reduced drug sensitivity, whereas negative correlations indicate potentially increased sensitivity. Drug mechanisms were assigned according to their principal reported or database-annotated targets. For investigational compounds with broad or incompletely characterized target profiles, the compounds were classified as experimental

kinase inhibitors or experimental targeted agents. These cell-line-based associations do not establish clinical drug response or causality.
