# Supplementary material for: Exploratory Multi-Platform Bioinformatic Analysis of MEAK7 and Its Molecular, Survival, and Immune Associations in Pancreatic Ductal Adenocarcinoma
Source: Int J Mol Sci. 2026 Aug 25;27(17):7610. doi: 10.3390/ijms27177610 (PMC13565933; doi:10.3390/ijms27177610)
Supplement: Supplementary file 1 [file ijms-27-07610-s001.zip › Supplementary Table S2.pdf]

Supplementary Table S2. Summary of the analytical platforms, input data, user-defined parameters, statistical methods, and outputs used in the study.

**Analysis**	**Platform**	**Input**	**User-defined parameters**	**Statistical analysis**	**Output**
Gene expression	GEPIA3	MEAK7 gene symbol	Default platform settings; $\|log2 fold change\| \geq 1$; $p < 0.05$	DESeq2-based differential-expression analysis; Wald test; Benjamini–Hochberg FDR correction	TPM expression in tumor and normal tissues
Protein expression	UALCAN (CPTAC)	MEAK7 gene symbol	Sex and mTOR-pathway-status subgroup analyses; normal pancreatic tissue used as the reference group	Two-sample Student's t-test	Total protein expression levels
Protein expression	Human Protein Atlas	MEAK7 gene symbol	Normal pancreas and pancreatic cancer tissue selection; HPA041020 antibody; platform-provided staining categories	Descriptive analysis; no independent image quantification or inferential testing	Immunohistochemical staining images
Differential expression	TNMplot	MEAK7 gene symbol	Tumor vs. normal; metastatic vs. primary;	Kruskal–Wallis and Dunn's multiple comparisons	Scaled expression values and adjusted *p* values
Co-expression analysis	TNMplot	MEAK7 gene symbol	Pancreatic tumor samples	Spearman's rank correlation with Benjamini–Hochberg FDR correction	Ranked genes, Spearman's ρ, nominal *p* values, and FDR-adjusted *p* values

Analysis	Platform	Input	User-defined parameters	Statistical analysis	Output
Differential expression	GEO2R (GSE62165, GSE71729, GSE183795)	MEAK7 probe(s)	Dataset-specific group definitions; $\lvert log2FC \rvert > 1$; FDR-adjusted $P < 0.05$; representative probe selected according to the lowest adjusted P value when multiple probes mapped to MEAK7/KIAA1609	Limma with Benjamini–Hochberg FDR correction	LogFC, adjusted *P* value
Exploratory ROC-based tissue-expression discrimination	Jamovi 2.3.28	Normalized and batch-corrected GSE62452 expression matrix; 69 PDAC tumor and 61 adjacent non-tumor pancreatic tissue samples; MEAK7, KRAS, TP53, CDKN2A, and SMAD4	Tumor tissue defined as the positive class; adjacent non-tumor tissue defined as the negative class; gene-specific cutoff maximizing the Youden index	ROC analysis; nonparametric DeLong standard error; two-sided 95% CI calculated as AUC ± 1.96 × SE_DeLong	AUC with 95% CI, Youden-optimal cutoff, sensitivity, and specificity
Survival analysis	Kaplan–Meier Plotter	MEAK7 gene symbol	Overall survival and disease-free survival; auto-select best cutoff	Kaplan–Meier analysis,<br>Cox proportional hazards model and log-rank test	Survival curves, hazard ratio, 95% CI, log-rank P value, FDR-adjusted p value, and median survival time
Survival analysis	DoSurvive	MEAK7 gene symbol	Overall survival and disease-free interval; median expression stratification; adjustment	Kaplan–Meier analysis and multivariable Cox	Hazard ratios, 95% CIs, and *p* values

Analysis	Platform	Input	User-defined parameters	Statistical analysis	Output
			for age, sex, and tumor stage	proportional hazards regression	
Immune infiltration	TIMER 3.0	MEAK7 gene symbol	Selected immune infiltrates; tumor-purity adjustment	Tumor-purity-adjusted partial Spearman correlation with Benjamini–Hochberg FDR correction	Correlation coefficient (ρ), nominal P value, and FDR-adjusted P value
Immune infiltration–stratified survival	TIMER 3.0	MEAK7 gene symbol	OS and DSS; Group 4 vs. Group 3; selected immune/stromal components	Kaplan–Meier analysis with Benjamini–Hochberg FDR correction	Hazard ratio and nominal/adjusted *P* value
miRNA target prediction	TargetScanHuman 8.0 and miRDB	MEAK7 gene symbol	miRDB target score ≥80	Computational target-prediction algorithms	Candidate and overlapping MEAK7-targeting miRNAs
miRNA correlation	ENCORI	MEAK7 and selected genes	TCGA-PAAD cohort; default expression settings	Platform-reported correlation coefficient (r), P value, and linear regression	Co-expression coefficients and regression plots
Pancreatic cancer–associated lncRNA identification	LncRNADisease	“Pancreatic cancer” disease term	Experimentally supported disease-associated lncRNAs	Curated evidence retrieval/descriptive analysis	Pancreatic cancer–associated lncRNAs and supporting annotations
lncRNA correlation	ENCORI	MEAK7 and selected genes	TCGA-PAAD cohort; default expression settings	Platform-reported correlation coefficient (r), P value, and linear regression	Co-expression coefficients and regression plots

**Analysis**	**Platform**	**Input**	**User-defined parameters**	**Statistical analysis**	**Output**
Functional association network	STRING v12.0	MEAK7 gene symbol	first-shell interactors; minimum interaction score ≥0.700 (high confidence); edges supported by experimental evidence or curated databases	STRING confidence scoring	Functional association network, evidence channels, and combined confidence scores
Exploratory machine-learning–based tissue classification	Orange Data Mining v3.40.0	Normalized and batch-corrected GSE62452 expression matrix; prespecified 11-gene MEAK7-centered STRING-derived panel; 69 PDAC tumor and 61 adjacent non-tumor samples, including 60 matched pairs and 10 unmatched samples	Tumor tissue defined as the positive class; fixed pair-aware five-fold cross-validation based on CV_Fold; paired samples retained in the same fold; training-fold standardization to $\mu=0$ and $\sigma^2=1$; no outcome-guided feature selection or post hoc hyperparameter optimization	Logistic regression, Random Forest, Gradient Boosting, SVM, Tree, kNN, and Neural Network; performance estimated from aggregated out-of-fold predictions	AUC, CA, F1 score, precision, sensitivity, specificity, MCC, ROC curves, confusion matrices, and graphical workflow
Transcriptomic assessment in pancreatic cancer cell lines	EMBL-EBI Expression Atlas	MEAK7 gene symbol	Pancreatic adenocarcinoma; Homo sapiens; cell-line datasets only	Descriptive platform-based expression assessment	Platform-defined low, medium, and high expression categories
In silico gRNA prioritization	CRISPRdb	MEAK7 gene symbol	Default platform settings	Predicted on-target potency scoring and computational off-target classification	Candidate gRNA sequences, potency scores, and categorical off-target annotations

Analysis	Platform	Input	User-defined parameters	Statistical analysis	Output
Drug sensitivity	GSCA	MEAK7 gene symbol	GDSC and CTRP datasets; FDR ≤ 0.05	Pearson correlation analysis with false discovery rate correction	Correlation coefficients between MEAK7 expression and drug sensitivity

The analytical workflow used in this study is summarized in Supplementary Table S2. For web-based platforms, reproducibility was supported by reporting the user-accessible analytical parameters, statistical procedures, database versions, access dates, and relevant methodological references. For the investigator-run analyses, the GSE62452 analysis matrix containing the fixed CV_Fold assignments, the jamovi analysis file (.omv), and the Orange workflow file (.ows) were submitted to the editor and reviewers as files for review only. A graphical representation of the Orange workflow is provided as Supplementary Figure S1 in scalable vector graphics format.
